# Long-term real-world evidence of sparsentan efficacy in patients with IgA nephropathy treated with SGLT2 inhibitors

**DOI:** 10.1093/ckj/sfag181

**Published:** 2026-06-01

**Authors:** Moritz Schanz, Claudia Seikrit, Bernd Hohenstein, Aline Zimmermann, Leonie Kraft, Severin Schricker, Andrea Schwab, Tina Oberacker, Joerg Latus

**Affiliations:** Department of General Internal Medicine and Nephrology, Robert Bosch Hospital Stuttgart, Stuttgart, Germany; Division of Nephrology and Clinical Immunology, RWTH Aachen University Hospital, Aachen, Germany; Nephrological Center Villingen-Schwenningen, Villingen-Schwenningen, Germany; Dialysis Center of the Arbeitsgemeinschaft Heimdialyse Saar e.V., Homburg, Germany; Department of General Internal Medicine and Nephrology, Robert Bosch Hospital Stuttgart, Stuttgart, Germany; Department of General Internal Medicine and Nephrology, Robert Bosch Hospital Stuttgart, Stuttgart, Germany; Department of General Internal Medicine and Nephrology, Robert Bosch Hospital Stuttgart, Stuttgart, Germany; Dr Margarete Fischer-Bosch-Institute of Clinical Pharmacology and University of Tuebingen, Stuttgart, Germany; Department of General Internal Medicine and Nephrology, Robert Bosch Hospital Stuttgart, Stuttgart, Germany

**Keywords:** dual endothelin angiotensin receptor antagonist (DEARA), IgA nephropathy, real-world data, sodium–glucose cotransporter 2 (SGLT2) inhibition, sparsentan

## Abstract

**Background:**

Sparsentan has emerged as a promising therapeutic option for immunoglobulin A nephropathy. However, data on its effectiveness in real-world settings, particularly in combination with sodium–glucose cotransporter 2 (SGLT2) inhibitors remain limited. In our previously published real-world cohort (*n* = 23), we demonstrated a significant reduction in proteinuria with concurrent SGLT2 inhibitor treatment. Here, we present 1-year follow-up data.

**Methods:**

After 1 year, 17 patients (74%) from the initial cohort remained on sparsentan therapy. As described previously, patients had been on stable, maximally tolerated renin–angiotensin system (RAS) inhibition and stable SGLT2 inhibitor therapy before replacement of RAS inhibition with sparsentan; eligibility required an estimated glomerular filtration rate (eGFR) >30 ml/min/1.73 m^2^ and a urine protein/creatinine ratio (UPCR) >0.75 g/g.

**Results:**

Baseline median (interquartile range) eGFR (Chronic Kidney Disease Epidemiology Collaboration) was 48 ml/min/1.73 m^2^ (34–66) and median UPCR was 1.55 g/g (0.90–1.85). At 1-year follow-up, UPCR remained significantly reduced (*P* = .0001) to a median of 0.54 g/g (0.34–0.76), corresponding to a relative reduction of 61% (45–95). The median chronic eGFR slope was −1.5 ml/min/1.73 m^2^ per year (−7.51 to +3.75) at follow-up.

**Conclusions:**

In this 1-year follow-up of our real-world cohort, sparsentan was associated with a significant and sustained reduction in proteinuria over 12 months, with a numerically less steep eGFR decline during follow-up, even in patients already receiving SGLT2 inhibitors.

## Graphical Abstract

**Graphical Abstract:**
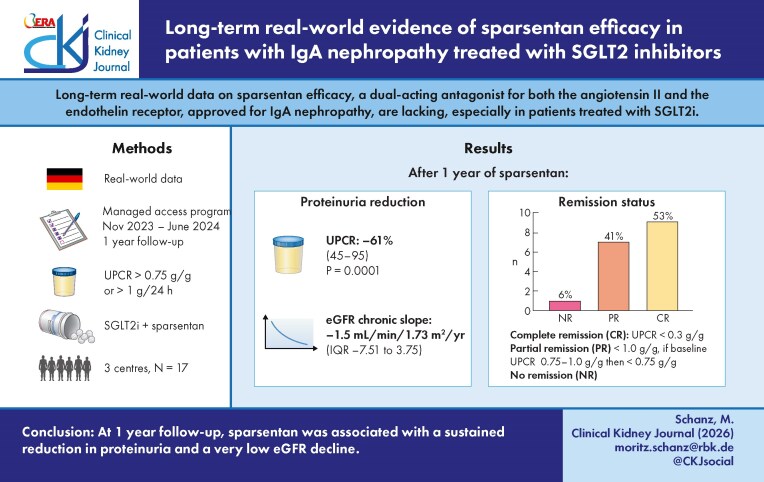

KEY LEARNING POINTS **What was known:**

- Sparsentan, a dual-acting antagonist for both the angiotensin II receptor type 1 and the endothelin receptor type A, has been recently approved for the treatment of immunoglobulin A nephropathy (IgAN), exhibiting significant efficacy in reducing proteinuria and slowing down IgAN progression.
- Information and real-world data on long-term effects on top of pre-existing sodium–glucose cotransporter 2 (SGLT2) inhibition are lacking.

**This study adds:**

- Sparsentan demonstrated additional and long-term effects over 12 months on reducing proteinuria and achieving complete remission in patients who were on SGLT2 inhibitors in a real-world setting.
- The antiproteinuric effect was accompanied by a less steep estimated glomerular filtration rate slope and largely stable kidney function over 1 year, with a favourable safety profile in routine clinical practice.

**Potential impact:**

- These data support sparsentan as a practical backbone therapy within the chronic kidney disease management pillar of the 2025 Kidney Disease: Improving Global Outcomes (KDIGO) IgAN framework, even in patients already receiving SGLT2 inhibitors.

## INTRODUCTION

Immunoglobulin A (IgA) nephropathy (IgAN) is the most frequent primary glomerular disease worldwide and is one of the major causes of kidney failure in young adults [1]. Usually, IgAN formerly was regarded as a kidney disease with relatively benign course, but recent population-based data from large observational registers such as German Chronic Kidney Disease (GCKD) cohort or UK National Registry of Rare Kidney Diseases (RaDaR) have questioned this assumption, demonstrating that even patients with proteinuria under 1 g/day experience progressive kidney function loss and long-term kidney failure, and have challenged the previously declared ‘safe threshold’ of proteinuria. This underscores that IgAN is rarely an indolent disease [1–3].

The pathophysiology of IgAN is represented in the ‘multihit hypothesis’, which involves pathogenic relevant circulating IgA of probably mucosal origin that leads to IgA/IgG immune complex formation and deposition within the renal mesangium, leading to chronic glomerular inflammation and progressive kidney damage [4]. Despite this mechanistic insight, the heterogeneity of the underlying immune and inflammatory processes and the hurdle that previous conventional trial endpoints were not useful in this rare disease have complicated the development of disease-specific therapies. Through identification of surrogate parameters, such as proteinuria reduction and the rate of (estimated) glomerular filtration rate (eGFR) decline (eGFR slope), and its acceptance by the regulatory authorities, a new era in drug development for IgAN has begun [5–7].

Until recently, the treatment of IgAN was based almost entirely on optimized supportive care. Rigorous blood pressure control, renin–angiotensin system inhibition (RASi) at the maximum tolerated dose, and lifestyle modification formed the cornerstone of therapy [8]. In the meantime, sodium–glucose cotransporter 2 inhibitors (SGLT2i) have now become part of the standard of care for chronic kidney disease (CKD) patients, including those with IgAN, given their consistent nephroprotective effects and risk reduction in CKD progression across etiologies [9, 10].

Conventional immunosuppressive therapy with systemic corticosteroids remains controversial due to its severe toxicity, as demonstrated in the TESTING trial [11]. More selective approaches with targeted-release formulation (TrF)-budesonide offered—even though not without side effects—a safer gut-directed corticosteroid approach; however, treatment effects appeared to diminish after discontinuation [4, 12].

The revised 2025 Kidney Disease: Improving Global Outcomes (KDIGO) guideline on the management of IgAN, released in October 2025, now provides a structured framework for IgAN treatment, discriminating two therapy pillars [13]: (i) CKD management—focusing on nephroprotection and controlling the consequences of existing IgAN-induced nephron loss through optimized supportive measures, RAS blockade, and SGLT2 and endothelin A inhibition; and (ii) IgAN-specific therapy—by preventing or reducing IgA-containing immune complex (IgA-IC) formation and IgA-IC-mediated glomerular injury via immunomodulatory or anti-inflammatory interventions.

Both pillars are intended to be addressed simultaneously, depending on the patient’s individual risk of disease progression [13].

Among the novel therapeutic developments, sparsentan, a dual endothelin type A and angiotensin II receptor antagonist (DEARA), represents the first nonimmunosuppressive drug approved for the treatment of IgAN and is recommended by the KDIGO guideline [13], based on the data from the PROTECT trial [12, 14]. Although primarily considered as a CKD-directed therapy to reduce IgAN-related nephron loss, emerging evidence points towards additional immunomodulatory and anti-inflammatory effects: Interim data from the phase II SPARTAN trial showed a 50% reduction in urinary CD163, alongside a 75% decrease in urinary B cell activating factor (BAFF), and a 68% decrease in C5b9, suggesting a downregulation of macrophage activation, B-cell signaling, and complement activation pathways [15].

Sparsentan became available in Germany in August 2024, following its approval in Europe earlier that year, and was accessible beforehand through a managed access program (MAP) for high-risk IgAN patients starting end of 2023 [16, 17]. In this context, we have previously reported short-term real-world outcomes confirming its clinical efficacy and tolerability, even in combination with stable SGLT2i therapy—an important observation since concomitant SGLT2i use was not permitted in the PROTECT trial [18].

Our study aims to extend our previous findings by analyzing 1-year follow-up data of all patients treated within the MAP program, focusing on the long-term effectiveness—on top of SGLT2i effect—of sparsentan in a real-world clinical setting.

## MATERIALS AND METHODS

### Study design and population

IgAN patients were treated with sparsentan during clinical routine from November 2023 through the MAP prior to the official approval. In this retrospective multicentre analysis, we provide real-world evidence on the long-term efficacy and safety of sparsentan in patients with IgAN during clinical routine.

The study was conducted in the private practice sector but also outpatient clinics from referral centres across several nephrology centres in Germany: involved centres were the Robert Bosch Hospital in Stuttgart, the Nephrological Center in Villingen-Schwenningen, and the Dialysis Center of the Arbeitsgemeinschaft Heimdialyse Saar e.V. in Homburg.

**Figure 1:**
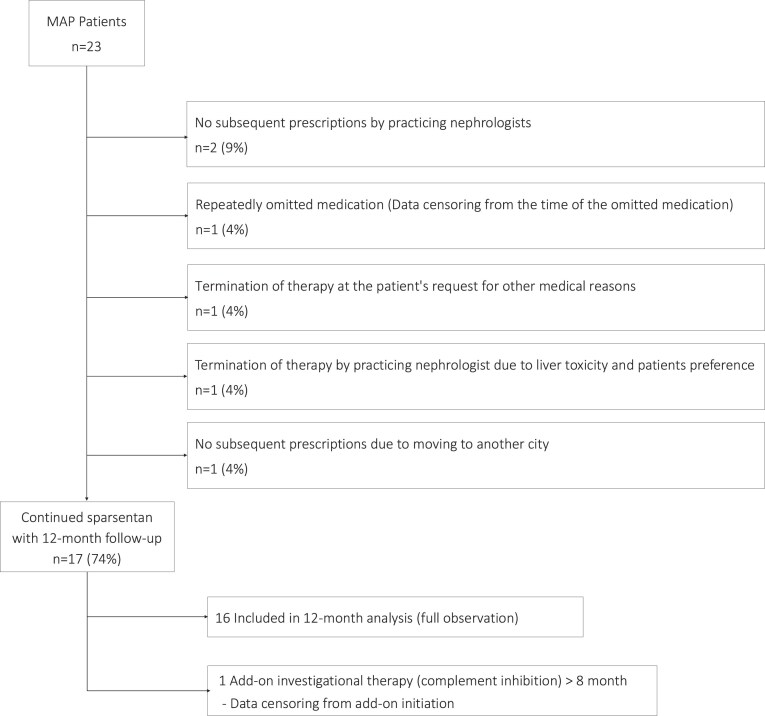
Study flow diagram showing reasons for not continuing sparsentan at 1-year follow-up. M: month; Y: year.

All patients who participated in the MAP were screened. Analysis of the 12-month on-treatment outcomes focused on patients who remained on sparsentan at 12 months; reasons for discontinuation are shown in Fig. 1. The initial study aimed to include all patients of the MAP, ending by June 2024, prior to the launch of sparsentan in August 2024. This follow-up analysis 12 months after sparsentan initiation continued up to until September 2025, when all patients reached the 12-month time frame. Target dose of sparsentan was 400 mg/day after 2 weeks of initial treatment at the half dose according to the MAP study protocol.

Inclusion criteria for our analysis were patients who were on stable SGLT2i therapy (at least >3 months) and participating in the sparsentan MAP as already described previously [18]. Briefly, inclusion criteria for the MAP were as follows: (i) stable dose of maximal tolerated angiotensin-converting enzyme (ACE) inhibitor and/or angiotensin receptor blockers (ARB) for at least 3 months prior to inclusion; (ii) eGFR ≥30 ml/min/1.73 m^2^ at eligibility review; (iii) urine protein/creatinine ratio (UPCR) ≥0.75 g/g or urine protein excretion value ≥1.0 g/day; (iv) mean seated blood pressure of ≥100/60 and ≤160/100 mmHg; (v) aged ≥18 years; and (vi) women of childbearing potential had to use effective methods of contraception.

Exclusion criteria were as follows: (i) IgAN secondary to another condition; (ii) enrolment in an interventional clinical trial; (iii) concomitant use of any of renin–angiotensin–aldosterone system (RAAS)/endothelin receptor (ERA) antagonists, strong cytochrome P450, family 3, subfamily A (CYP3A) inhibitors or inducers, and potassium-sparing drugs; (iv) undergone organ transplantation; (v) documented heart failure New York Heart Association (NYHA) II–IV or unexplained dyspnoea; (vi) clinically significant cerebrovascular/coronary artery disease within 6 months prior to inclusion; (vii) hepatobiliary disease; (viii) anaemia (haemoglobin <9 g/dl); (ix) potassium values >5.5 mmol/l; (x) hypersensitivity/allergic response to ARB or ERA; (xi) pregnancy/breastfeeding; and (xii) nonadherence in the opinion of the physician.

All patients provided written informed consent for data collection and analysis as part of the MAP. This study was conducted with consultation of the ethics committee of the University of Tuebingen, Germany (384/2024BO2).

### Data collection

Collected data included baseline medication, previous immunosuppressive therapy, and pre-existing conditions. Primary data sources were patient reports, including laboratory values from outpatient care, records with collected data from the MAP, the hospital information system, and the digital archive. No study-associated investigations were conducted; only routine parameters were recorded.

Whenever available, the following data were included: weight, blood pressure, laboratory values, including basic internal laboratory results such as red blood cell (RBC) count, eGFR, Aspartate aminotransferase (AST), alanine aminotransferase (ALT), gamma-glutamyl transferase (GGT), bilirubin, electrolytes, serum albumin, haematuria, proteinuria, and microalbuminuria, kidney biopsy details, such as Oxford classification [19] and interstitial fibrosis and tubular atrophy (IF/TA) grading [20], and all reported adverse events (AEs).

### Treatment and Follow-up

In the context of MAP, RASi were discontinued 48 h, and mineralocorticoid receptor antagonists (MRAs) were stopped 1 week prior to sparsentan initiation.

Follow-up visits were conducted as part of routine care, and available data were analyzed at ∼6, 9, and 12 months after sparsentan initiation. Appointments outside these windows were assigned to the nearest scheduled follow-up time point.

### Definitions

Treatment response was adapted from the PROTECT study criteria at any time during treatment in the observation period and we only focused on proteinuria (haematuria was not taken into account). As no data on 24-h urine collection were available [14], response was categorized as follows: partial remission (PR): proteinuria <1.0 g/g, if baseline was between 0.75 and 1.0 g/g, then <0.75 g/g; complete remission (CR): proteinuria <0.3 g/g; and no remission (NR), if neither of both criteria were met. If only urine albumin/creatinine ratio (UACR) was available, PR was defined correspondingly with UACR <1000 mg/g and CR with UACR <300 mg/g. We additionally report the proportions of patients achieving ≥30% or ≥50% reduction in UPCR or UACR, following the approach used in the ongoing SPARTACUS trial [21]. Haematuria was not used for the definition of remission to maintain comparability with the PROTECT trial, where remission was defined based solely on proteinuria. In addition, because sparsentan is primarily positioned within the CKD management pillar of the current KDIGO framework, a consistent effect on haematuria was not expected.

Haematuria was analyzed automatically as part of the routine measurement and partly based on dipstick testing (RBCs/microlitre). Haematuria of >28 RBCs/μl were considered positive according to previous work [22]. Obesity was defined according to World Health Organization with a body mass index (BMI) ≥30 kg/m². Groups for subgroup analysis were divided at median or otherwise clinically indicated.

### eGFR slope calculations

Individual eGFR slopes were estimated by simple linear regression using all available eGFR values within the 12 months before sparsentan initiation. The chronic on-treatment eGFR slope was estimated using all available eGFR values obtained from week 6 onwards. A minimum of three measurements per patient was required for regression-based slope estimation; in patients with only two historical values, a linear slope was derived from these two time points. All available measurements within the defined time windows were included without imputation. Included and excluded patients for historic slope analysis are shown in Table S2. For historic slope calculations, patients had a median of 4 [interquartile range (IQR) 3–5] eGFR measurements, covering a median time span of 9 (5.25–11.00) months, with a median look-back period of 11 (7.25–13.00) months.

### Statistical analysis

For group comparisons of baseline characteristics, categorical variables were analyzed using the Fisher exact and chi-square test and of continuous variables using the *t*-test and Mann–Whitney test, respectively, for normally and non-normally distributed variables. For paired nonparametric variables, Wilcoxon matched-pairs signed rank test was used. Percentage reduction in proteinuria was calculated as the median of individual patient-level percentage changes. Statistical analysis was performed using Prism (Version 10.5.0, GraphPad, Software Inc., La Jolla, USA).

## RESULTS

For the MAP, a total of 121 patients with biopsy-proven IgAN were screened for eligibility. Of these, 98 patients were excluded due to exclusion criteria (55%), lack of availability/response (23%), or refusal to participate (3%). The remaining 23 patients were treated with sparsentan and included in the initial analysis of clinical outcomes and reported previously [18]. After 1 year, *n* = 17 patients (74%) from the initial cohort remained on sparsentan therapy and were included in our long-term analysis. The reasons for not continuing sparsentan in the six patients at follow-up are shown in Fig. 1. These 17 patients constituted the on-treatment follow-up (FU) cohort. Twelve-month outcome data were available for 16 of these 17 patients (94%); one patient entered a complement inhibitor trial after 8 months while continuing sparsentan.

All patients started sparsentan at half dose and reached the target dose of 400 mg/day after 2 weeks according to the MAP protocol.

No significant differences in baseline characteristics were observed between noncompleters (*n* = 6) of the original cohort (*n* = 23) and completers (*n* = 17) (see Table S1).

### Baseline characteristics

Baseline demographic and clinical characteristics of the initial cohort (*n* = 23) and the 12-month FU cohort (*n* = 17) are summarized in Tables 1 and 2 . Patients in the FU cohort were representative of the initial population.

**Table 1:** Baseline characteristics at sparsentan initiation.

	Initial cohort (*n* = 23)	FU cohort (*n* = 17)	*P*-value
	Median (IQR)		
Age at sparsentan initiation (y)	38 (26–48)	38 (27–47)	.99
Female gender (*n*, %)	10 (44)	8 (47)	>.99
Race			
White (*n*, %)	21 (91)	15 (88)	>.99
Asian (*n*, %)	2 (9)	2 (12)	
Time from initial kidney biopsy to sparsentan initiation (m)	34 (10–55)	18 (6–40)	.22
BMI (kg/m^2^)	26.8 (24.0–30.9)	24.8 (22.2–26.2)	.44
Blood pressure syst. (mmHg)	130 (125–135)	128 (123–138)	.78
Blood pressure diast. (mmHg)	80 (76–90)	80 (75–88)	.66
Comorbidities	N (%)		
Arterial hypertension	15 (65)	10 (59)	.75
Hyperlipoproteinaemia	10 (44)	7 (41)	>.99
Diabetes mellitus	2 (9)	1 (6)	>.99
Obesity	6 (26)	2 (12)	.43
Medication	N (%)		
ACE inhibitors/ARB^a^ (RASi)	23 (100)	17 (100)	>.99
ACE inhibitor or ARB at maximum labelled dose at screening	17 (74)	11 (65)	.73
MRA^a^	1 (4)	0 (0)	>.99
SGLT2 inhibitors	23 (100)	17 (100)	
Dapagliflozin (10 mg)	21 (91)	15 (88)	>.99
Empagliflozin (10 mg)	2 (9)	2 (12)	
History of corticosteroid therapy	13 (57)	10# (59)	>.99
Systemic	5 (22)	4 (24)	>.99
Ongoing^b^	0 (0)	0 (0)	
<12 months^b^	0 (0)	0 (0)	
TrF-budesonide	8^+^ (35)	7 (41)	.75
Ongoing^b^	4 (17)	4 (24)	
<12 months^b^	3 (13)	3 (18)	
Diuretics	8 (35)	5 (29)	>.99
Calcium channel blockers	10 (44)	7 (41)	>.99
Beta blockers	3 (13)	1 (6)	.62
Other antihypertensive therapy agents	6 (26)	4 (24)	.71
Lipid lowering therapy	12 (52)	8 (47)	>.99

a Discontinued prior to sparsentan initiation. ^+^One patient received classic budesonide.

b Ongoing: overlapping therapy with sparsentan (for detailed description see Fig. S1); <12 months: therapy within 12 months before sparsentan initiation, but not overlapping.

c One patient received ongoing TrF-budesonide with history of systemic corticosteroid history.

BL: baseline; Y: year.

**Table 2:** Baseline renal parameters at sparsentan initiation.

Baseline renal parameters	Initial cohort (n = 23)	FU cohort (n = 17)	*P*-value
	Median (IQR)		
Baseline eGFR (CKD-EPI) (ml/min/1.73 m^2^)	42 (32–63)	48 (34–66)	.71
eGFR decline in the preceding year (ml/min/1.73 m^2^ per year)			
Excluding patients on corticosteroids	−2.82 (−12.0 to +0.5)	−3.00 (−19.14 to +6.23)	.92
Patients on corticosteroids	+6.3 (+1.9 to +9.2)	+6.69 (+1.09 to +14.87)	>.99
eGFR decline under sparsentan therapy (chronic slope)	-	−1.51 (−7.51 to +3.75)	
eGFR category (CKD-EPI) (n, %)			
≥90 ml/min per 1.73 m^2^	3 (13)	2 (12)	>.99
≥60 to <90 ml/min/1.73 m^2^	4 (17)	3 (18)	>.99
≥45 to <60 ml/min/1.73 m^2^	4 (17)	4 (24)	.70
≥30 to <45 ml/min/1.73 m^2^	10 (44)	7 (41)	>.99
≥15 to <30 ml/min/1.73 m^2#^	2 (9)	1 (6)	>.99
Serum albumin (g/dl)	3.9 (3.7–4.2)	3.9 (3.7–4.2)	.83
Haematuria (*n*, %)	16 (70)	14 (82)	.47
Baseline UPCR (g/g)	1.5 (0.9–1.8)	1.55 (0.90–1.85)	.79
Baseline UACR (mg/g)	1254 (786–1665)	934 (675–1719)	.71

a eGFR progress <30 ml/min/1.73 m^2^ between screening and sparsentan initiating.

**Table 3:** Histological characteristics/Oxford MEST-C scoring system (*n*, %).

Score	M (0, 1)	E (0, 1)	S (0, 1)	T (0, 1, 2)	C (0, 1, 2)	IF/TA category	fsGS category	gGS category
0	3 (18)	14 (82)	0 (0)	8 (47)	12 (71)	2 (12)	0 (0)	1 (6)
1	12 (71)	1 (6)	15 (88)	5 (29)	4 (24)	9 (53)	10 (59)	6 (35)
2	–	–	–	2 (12)	0 (0)	4 (24)	3 (18)	6 (35)
3	–	–	–	–	–	2 (12)	2 (12)	2 (12)
Not stated/possible	2 (12)	2 (12)	2 (12)	2 (12)	1 (6)	0 (0)	2 (12)	2 (12)

Definition IF/TA, fsGS, gGS category: 0: absent; 1 (mild): <25%; 2 (moderate): 25%–50%; and 3 (severe): >50% of the total area/of the glomeruli.

M: Mesangial hypercellularity; E: Endocapillary hypercellularity; fsGS: Focal-segmental glomerulosclerosis; gGS: Global glomerulosclerosis; S: Segmental sclerosis; T: Interstitial fibrosis/tubular atrophy (Oxford MEST-C); C: Crescents. IF/TA: Interstitial fibrosis and tubular atrophy.

At sparsentan initiation, median age in the FU cohort was 38 years (IQR 27–47), 47% were female, and the vast majority were White (88%) or Asian (12%). Median BMI was 24.8 kg/m² (22.2–26.2). Median time from initial diagnostic kidney biopsy to treatment initiation was 18 months (6–40).

The most frequent comorbidities were arterial hypertension (59%), hyperlipoproteinaemia (41%), and obesity (12%); one patient (6%) had diabetes mellitus. All patients received a maximally tolerated RASi (ACE inhibitor or ARB) before switching to sparsentan, and all were on stable SGLT2i therapy for at least 3 months at baseline. Two-thirds (65%) were on the maximum labelled dose of RASi. A history of systemic corticosteroid or TrF-budesonide use was present in 59% of patients, and four patients (24%) were still on TrF-budesonide at screening (for detailed breakdown see Table S3 and Fig. S1).

Median baseline eGFR [Chronic Kidney Disease Epidemiology Collaboration (CKD-EPI)] was 48 ml/min/1.73 m^2^ (34–66), with a balanced distribution across CKD stages and a predominance of patients in KDIGO G3b (≥30 to <45 ml/min/1.73 m^2^, 41%) (Table 2). Median baseline UPCR was 1.55 g/g (0.90–1.85), and median UACR was 934 mg/g (675–1719). Haematuria (>28 RBCs/μl) was present in 82% of patients.

In the year prior to sparsentan initiation, the median annual eGFR decline was −3.00 ml/min/1.73 m^2^ (−19.14 to +6.23) (excluding patients receiving corticosteroids including TrF-budesonide due to confounding effects on eGFR).

### Histological characteristics/Oxford MEST-C scoring

Histopathologic findings according to the Oxford MEST-C score and additional chronicity indices are shown in Table 3. Mesangial hypercellularity (M1) was present in the majority of patients (71%), whereas endocapillary hypercellularity (E1) was uncommon (6%), and crescents (C1/C2) were present in a minority (24%). Segmental glomerulosclerosis (S1) was observed in 89%. Interstitial fibrosis and tubular atrophy (T) were absent (T0; 47%) or mild (T1; 29%) in most patients, with only a small fraction (12%) showing moderate changes (T2).

Regarding focal-segmental glomerulosclerosis (fsGS) and global glomerulosclerosis (gGS), most patients had mild (59%/35%) to moderate (18%/35%) chronic damage, consistent with a cohort at high risk of progression but not yet in end-stage remodelling.

### Proteinuria reduction and remission status

**Figure 2:**
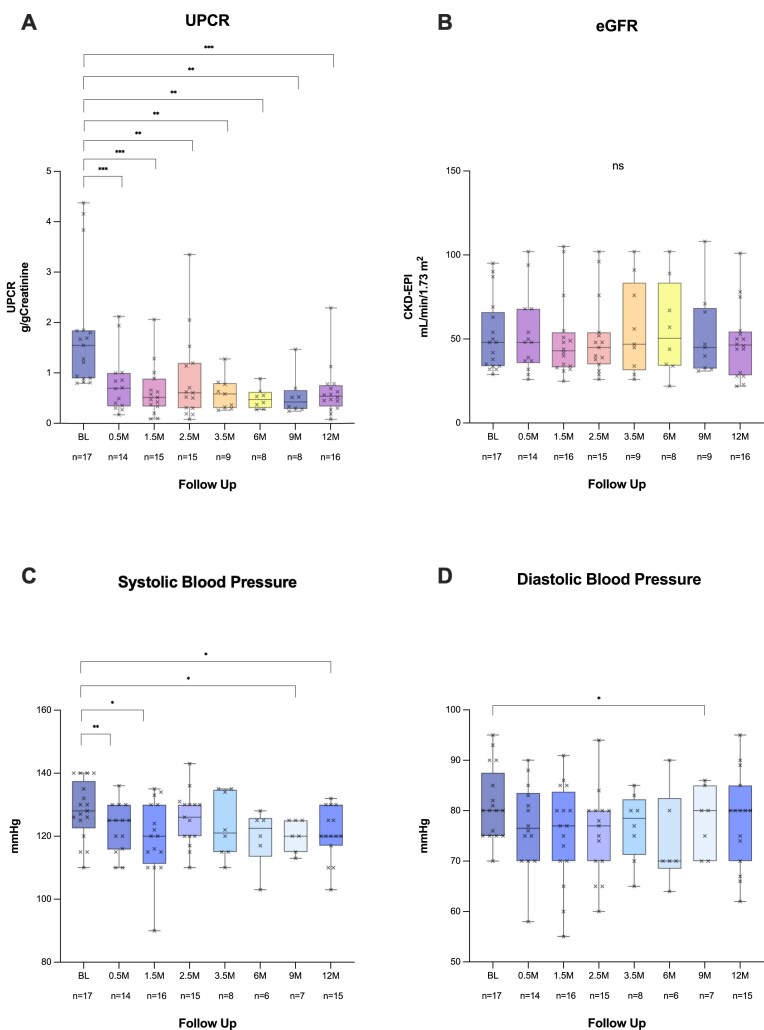
Course of proteinuria (UPCR) (A), eGFR (B), systolic (C), and diastolic blood pressure (D) under therapy with sparsentan. Box: median, IQR; Whisker: min–max. BL: baseline; M: month.

Sparsentan treatment led to a significant and sustained reduction in proteinuria over 12 months (Table 4 and Fig. 2A): in the FU cohort (*n* = 17), median baseline UPCR was 1.55 g/g (0.90–1.85), which decreased to 0.47 g/g (0.30–0.62) at 6 months, corresponding to a 68% (54–77) relative reduction versus baseline (*P* = .008), 0.42 g/g (0.29–0.66) at 9 months, a 65% (43–74) reduction (*P* = .008), and 0.54 g/g (0.34–0.76) at 12 months, a 61% (45–95) reduction (*P* = .0001).

**Table 4:** Course of proteinuria (UPCR/UACR) under therapy with sparsentan.

	UPCR (g/g)	% Reduction	*n*	*P*-value^a^	UACR (mg/g)	% Reduction	*n*	*P*-value^a^
	Median (IQR)				Median (IQR)			
Baseline	1.55 (0.90–1.85)	Ref.	17	–	934 (675–1719)	Ref.	17	–
6 M	0.47 (0.30–0.62)	68 (54–77)	8	.008	351 (181–550)	72 (53–81)	8	.008
9 M	0.42 (0.29–0.66)	65 (43–74)	8	.008	297 (148–653)	62 (50–78)	6	.03
12 M	0.54 (0.34–0.76)	61 (45–95)	16	.0001	351 (185–634)	63 (50–80)	15	.0002

Intermediate follow-up data at 6 and 9 months were available only in a subset of patients due to nonstandardized visit schedules in routine care.

a Between baseline and follow-up (absolute results); Wilcoxon matched-pairs signed rank test.

BL: baseline.

Comparable changes were observed for UACR, which decreased from a median of 934 mg/g (675–1719) at baseline to 351 mg/g (181–550) at 6 months (−72%, 53–81; *P* = .008), 297 mg/g (148–653) at 9 months (−62%, 50–78; *P* = .03), and 351 mg/g (185–634) at 12 months (−63%, 50–80; *P* = .0002).

A similar pattern of UPCR reduction was observed after excluding patients with overlapping or recent (<12 months) corticosteroid therapy (all received TrF-budesonide), and even was present in the subgroup receiving corticosteroid therapy (Fig. S2).

Over 12 months, CR of proteinuria (UPCR <0.3 g/g) was observed in 9 of 17 patients (53%), PR in 7 of 17 (41%), while only 1 patient (6%) did not meet remission criteria (NR) (Fig. 4A). Accordingly, 94% of patients achieved at least PR at some time point during follow-up, and all patients reached at least a 50% proteinuria reduction at least once during the 12-month observation period (Fig. 4B).

### Time course of kidney function and other clinical variables

**Figure 3:**
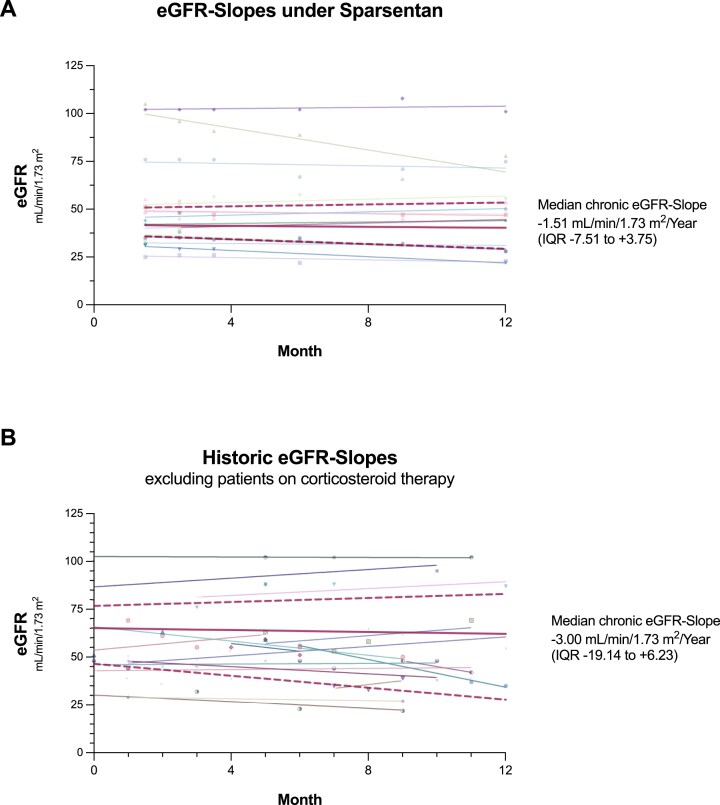
(A) Individual (thin lines) and median (bold line) [IQR (dotted lines)] chronic eGFR slopes over treatment period of 12 months with sparsentan and historic slopes (B) prior to sparsentan initiation. Patients who received corticosteroid therapy (targeted-release budesonide) during the historical slope period were excluded (*n* = 7), as treatment-associated increases in eGFR may confound slope estimation and limit its validity as a measure of underlying disease progression.

Median eGFR remained largely stable from 48 ml/min/1.73 m^2^ (34–66) at baseline to 47 ml/min/1.73 m^2^ (29–55) at 12 months (Fig. 2B). The annual median chronic eGFR slope under sparsentan therapy was −1.51 ml/min/1.73 m^2^ (−7.51 to +3.75) (Fig. 3 A), compared with -3.00 ml/min/1.73 m^2^ (−19.14 to +6.23) the pretreatment period, suggesting a numerically less steep decline (Fig. 3B). Median serum creatinine showed a small but statistically significant increase over 12 months (1.8 mg/dl at baseline vs 1.8 mg/d with a slightly higher upper IQR at 12 months, *P* = .04), paralleled by an increase in serum urea (51 vs 66 mg/dl, *P* = .0004) (Table 5).

**Figure 4:**
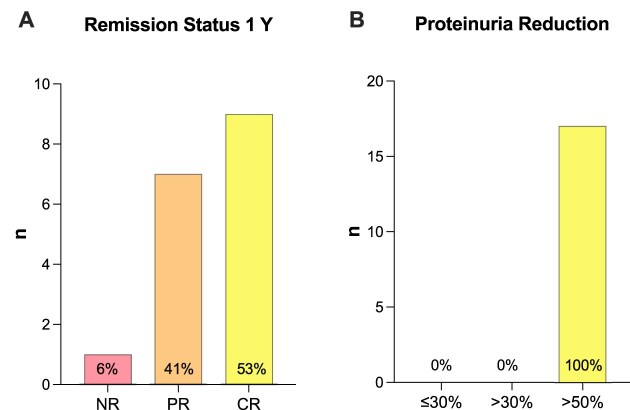
(A) Proportion of patients reaching CR/PR of proteinuria. Definition of proteinuria at least once at any time over the course of the treatment period: CR UPCR <0.3 g/g, PR is defined as <1.0 g/g, if baseline was between 0.75 and 1.0 g/g, then <0.75 g/g. (B) Proportion of patients with >30% or >50% UPCR reduction at any time during treatment in the observation period.

**Table 5:** Clinical and laboratory results at baseline before and after 12-month treatment with sparsentan.

	Baseline	12 months	*P*-value
Clinical variables Median (IQR)	*N* = 17		
Weight (kg)	74.8 (66.8–83.0)	77.0 (68.0–81.0)	.44
Blood pressure syst. (mmHg)	128 (123–138)	120 (117–130)	.01^a^
Blood pressure diast. (mmHg)	80 (75–88)	80 (70–85)	.21
Laboratory variables Median (IQR)			
eGFR (CKD-EPI) (ml/min/1.73 m^2^)	48 (34–66)	47 (29–55)	.05
Serum creatinine (mg/dl)	1.8 (1.3–2.1)	1.8 (1.3–2.3)	.04^a^
Urea (mg/dl)	51 (39–74)	66 (47–83)	.0004^a^
NTproBNP (pg/ml)	83 (38–132)	89 (47–143)	.99
Bilirubin (mg/dl)	0.4 (0.3–0.7)	0.5 (0.3–0.6)	.99
GOT/AST (U/l)	15 (14–21)	24 (17–28)	.13
GPT/ALT (U/l)	19 (13–25)	17 (13–23)	.38
GGT (U/l)	18 (14–24)	16 (14–25)	.31
Leucocytes (G/l)	7.7 (6.9–8.7)	6.7 (5.3–8.0)	.07
Haemoglobin (g/l)	134 (120–154)	133 (127–147)	.60
Platelets (G/l)	269 (221–322)	246 (196–278)	.003^a^
Potassium (mmol/l)	4.3 (3.9–4.6)	4.5 (4.3–4.9)	.08

a *P* < .05

BL: baseline.

Systolic blood pressure significantly decreased from a median of 128 mmHg (123–138) at baseline to 120 mmHg (117–130) at 12 months (*P* = .01), while diastolic blood pressure remained stable (Fig. 2C and D). Body weight did not change significantly (74.8 vs 77.0 kg, *P* = .44). Furthermore, other laboratory values like N-terminal pro-B-type natriuretic peptide (NTproBNP), liver enzymes (AST, ALT, GGT), bilirubin, leukocyte counts, and haemoglobin levels, were unchanged, suggesting no relevant hepatic or hematologic toxicity. Platelet counts decreased modestly from 269 to 246 G/l (*P* = .003), remaining within the normal range. Serum potassium rose slightly but nonsignificantly (4.3 to 4.5 mmol/l, *P* = .08) (Table 5).

### Haematuria

As described in the baseline characteristics, haematuria was present in the majority of patients at baseline (82%). During follow-up, no consistent or sustained reduction in haematuria across the entire cohort was remarkable. While individual changes occurred—especially in the extent of haematuria—there was no clear trend towards remission across all patients under sparsentan therapy. A decrease in haematuria was primarily observed in patients with prior or overlapping corticosteroid therapy. A detailed illustration of these changes can be found in Fig. S3.

### Subgroup analysis

Exploratory subgroup analyses were performed to identify factors associated with proteinuria reduction and remission at follow-up, acknowledging that the number of cases is small and that no definitive conclusions can be drawn.

First, we examined relative UPCR reduction (%) across various clinical and histologic subgroups, which were divided into two equal groups using the median or otherwise clinically indicated [e.g. gender (w/m) or steroid therapy (yes/no)]. Proteinuria reduction under sparsentan was consistent across most strata, including age (<38 vs ≥38 years), gender, baseline systolic blood pressure (<130 vs ≥130 mmHg), BMI (<25 vs ≥25 kg/m²), history or ongoing use of corticosteroids, baseline UPCR (<1.5 vs ≥1.5 g/g), and presence or absence of haematuria (Fig. S5A). No significant differences of the antiproteinuric effect were observed in any of these subgroups.

When stratified by histologic features, relative UPCR reduction was also similar across categories of mesangial hypercellularity (M0 vs M1), endocapillary hypercellularity (E0 vs E1), segmental glomerulosclerosis (S0 vs S1), and crescents (C0 vs C1/C2). However, patients with lower degrees of interstitial fibrosis and tubular atrophy (IF/TA <20%) showed greater proteinuria reduction compared with those with more advanced IF/TA (≥20%), suggesting that chronic tubulointerstitial damage may diminish the antiproteinuric effect. A similar trend was observed for lower gGS burden (Fig. S5B).

Second, we evaluated clinical and histologic factors associated with remission status over the 12 months. In this analysis, patients achieving CR (*n* = 9) were younger than those with PR or NR and tended to have higher baseline eGFR, lower BMI, less chronic histologic damage (lower gGS and IF/TA), and slightly lower baseline systolic blood pressure compared with nonremitters (Fig. S5C).

Additional exploratory subgroup analyses were performed to assess the proteinuria (UPCR) time course across clinically relevant strata. Overall, proteinuria reduction was observed across all subgroups, including patients with and without arterial hypertension, with and without prior corticosteroid therapy, across different baseline kidney function (eGFR ≥45 vs <45 ml/min/1.73 m^2^), and across degrees of chronic histological damage (Oxford T score T0 vs ≥T1) (Fig. S6).

While minor differences in the time course of proteinuria reduction were noted between subgroups, no consistent pattern indicating a differential treatment response was observed. These subtle differences are in line with the subgroup analyses presented above and may suggest a potentially attenuated response in patients with more advanced CKD. However, given the small sample size, these findings remain exploratory and should be interpreted descriptively.

### Safety

Sparsentan was generally well tolerated over 12 months. No drug-related serious AEs were observed. AEs considered possibly related to sparsentan included hypotension in two patients (12%), mild hyperkalaemia (≤5.5 mmol/l) in two (12%), oedema in two (12%), dizziness in one (6%), elevation of liver enzymes in one (6%) despite stable median values for the entire cohort, headache in one (6%), and pruritus in one (6%) (Table S4). One patient temporarily interrupted sparsentan due to symptomatic hypotension; treatment was successfully reintroduced and titrated to full dose without recurrence of symptoms. In one patient, therapy was discontinued by the treating outpatient physician due to elevated liver enzymes, and the patient was consequently not included in the 12-month analysis (Fig. 1).

Events judged unlikely to be related to sparsentan comprised one case of gout (6%) and one hospitalization for pneumonia (serious AE, 6%) (Table S4). Regarding SGLT2i therapy, no AEs involving urinary tract infections or genital fungal infections were observed. AEs were comparable between noncompleters (*n* = 6) of the original cohort (*n* = 23) and completers (*n* = 17), with no significant differences observed (Table S1).

## DISCUSSION

To our knowledge, this is the first real-world analysis reporting 12-month outcomes of sparsentan in IgAN on top of fully optimized supportive care including SGLT2i use and replacing maximally tolerated RAS blockade, interpreted in the context of the revised KDIGO guideline for treatment of IgAN [13]. In this early-use cohort, we observed an ∼60%–70% reduction in proteinuria over 1 year, a high rate of CR or PR, and a numerically less steep eGFR decline, with a safety profile consistent with—if not more favourable than—the pivotal trial data [12, 14].

### Antiproteinuric effect and kidney function preservation

The magnitude of proteinuria reduction under sparsentan in our cohort is remarkable: over 12 months, median UPCR and UACR were reduced up to 65% compared with baseline, and 94% of patients achieved at least PR, with more than half reaching CR. These findings are in line with, and in part exceed, the antiproteinuric effect reported in PROTECT [12, 14], despite our patients being treated in a heterogeneous real-world environment and all receiving concomitant SGLT2i therapy.

Data on the additive effects of sparsentan on top of SGLT2i are increasing: for example, early data from the phase II SPARTACUS trial showed a 56% reduction in UACR at week 24 with the addition of sparsentan to stable SGLT2i treatment [21]. While the SPARTACUS trial provides controlled prospective data on sparsentan in combination with SGLT2i background therapy, our study complements these findings by reflecting real-world routine clinical practice, including heterogeneous patient characteristics, nonstandardized monitoring, and longer follow-up.

These aspects may provide additional insight into real-world applicability beyond the trial setting.

While the sample size is small and the follow-up remains relatively short to draw firm conclusions on hard endpoints, the combination of a marked proteinuria reduction with largely stable eGFR over 12 months is consistent with a clinically meaningful nephroprotective signal and supports the potential role of sparsentan in the management of IgAN.

Overall, the class of endothelin receptor antagonists appears beneficial in IgAN: data on the selective endothelin-A receptor antagonist atrasentan from the phase III ALIGN trial showed that atrasentan significantly reduced proteinuria compared with placebo (−38% vs −3%; between-group difference −36%, *P* < .001) without any relevant increase of AEs or cases of fluid overload [23].

The temporal pattern also supports a mechanism beyond pure hemodynamics. As in our initial analysis, we see an early modest drop in systolic blood pressure followed by partial re-ascent towards baseline values, whereas the reduction in proteinuria remained stable. This argues against a purely blood-pressure driven effect and is consistent with preclinical data suggesting direct glomerular and podocyte-protective effects, potentially including anti-inflammatory mechanisms of DEARAs.

Despite discussion of potential anti-inflammatory effects, no consistent change in haematuria was observed in our cohort. This may reflect the primary positioning of sparsentan within CKD management, where effects on proteinuria and eGFR are more established, although the nonstandardized assessment of haematuria in this real-world cohort limits interpretability.

### Sparsentan in the KDIGO 2025 dual-pillar framework

The recently revised KDIGO guideline for IgAN treatment formalizes that therapy should target two synchronously addressed pillars [13]: CKD management and IgAN-specific therapy.

CKD management includes nephron protection, blood pressure control, and reduction of glomerular hyperfiltration (by lifestyle changes, RASi, SGLT2i, and endothelin pathway blockade), and IgAN-specific therapy includes modulation of the underlying immune and inflammatory processes (e.g. corticosteroids, TrF-budesonide, BAFF/a proliferation inducing ligand (APRIL) inhibitors, and complement-targeting strategies).

Within this framework, sparsentan is clearly positioned in pillar 1 (CKD management). Our 12-month real-world data strongly support this placement: on top of maximally tolerated RASi and SGLT2i, sparsentan led to further substantial reductions in proteinuria and stabilization of eGFR without introducing immunosuppressive toxicity. In other words, for patients with persistent proteinuria ≥0.5 g/day despite optimized supportive therapy, sparsentan appears to be the ‘default’ next step under the new guideline structure.

Signals from the SPARTAN trial suggest that sparsentan may act at the interface of both pillars [15]: primarily categorized as CKD therapy by KDIGO, but with relevant anti-inflammatory and immunomodulatory properties that may contribute to its disease-modifying effect.

Our data provide some early clues but do not fully answer this question. Subgroup analyses showed that the antiproteinuric effect of sparsentan was broadly consistent across clinical and histological strata. Younger age (which could also be due to adherence issues), and more advanced chronic histologic damage (especially IF/TA) tended to be associated with a lower probability of CR or proteinuria reduction, but not with a loss of response per se. In other words, most patients improved across all subgroups, while those with a more favourable baseline risk profile benefitted the most and were more likely to reach CR. This may suggest that earlier initiation of sparsentan after diagnosis could be associated with a greater likelihood of stabilizing kidney function; however, this observation remains hypothesis-generating.

From a guideline perspective, the current KDIGO approach remains deliberately pragmatic: sparsentan (on top of SGLT2i) is recommended for patients with evidence of progressive risk defined by proteinuria ≥0.5 g/day, although it should be noted that sparsentan is currently approved for UPCR ≥0.75 g/g in Europe.

Our own experience supports this concept of simultaneous treatment initiation. In our earlier analysis, we demonstrated that the concomitant use of sparsentan and TrF-budesonide appeared effective and safe, without an excess of AEs or loss of antiproteinuric efficacy [18]. The present 12-month follow-up—now reflecting clinical practice under the KDIGO 2025 framework—further suggests that CKD-pillar therapy with sparsentan can be maintained stably, while immunomodulatory treatments can be added or withdrawn depending on risk, activity, and tolerability. However, robust data on double or triple combinations (SGLT2i, sparsentan, and a dedicated immunosuppressive or immunomodulatory agent) are still sparse, and controlled studies addressing exactly this question are urgently needed.

Clinical variables and histology may help, but they are not sufficient to optimally guide combination therapy. A rational next step for the field will be prospective, biomarker-enriched studies that test whether selected profiles (e.g. high complement activation, high BAFF/APRIL, or persistent macrophage activation despite sparsentan) identify patients who benefit most from the addition of IgAN-specific immunomodulation on top of CKD-pillar therapy.

### Safety

Notably, we did not observe clinically relevant fluid retention or body weight increase. This contrasts with the fluid retention signal reported in SONAR trial with atrasentan in diabetic nephropathy and may reflect both patient cohort-specific differences and a possible protective effect of concomitant SGLT2i treatment, with natriuretic and osmotic diuretic properties [24–26]. Our findings are also consistent with long-term safety data from other sparsentan programs (e.g. DUET in fsGS), which showed sustained proteinuria reduction and stable safety profiles over several years of exposure [27].

### Strengths and limitations

However, our study has several limitations: first, the sample size was small, and not all patients completed the 12-month follow-up, which may limit statistical power. However, no significant differences in baseline characteristics or AEs were observed between completers and noncompleters, suggesting that attrition bias is unlikely to have materially affected the results. Second, the real-world design without a control group precludes direct comparison with other therapies and does not allow causal inference. Laboratory and clinical data collection, especially from private practice settings, was less standardized than in a trial environment, leading to some missing data. Third, slope analyses are limited by nonstandardized measurement intervals, potential nonlinearity of eGFR trajectories, and incomplete data on historic eGFR values due to the real-world setting. Fourth, safety data were collected pragmatically and not through predefined AE reporting forms, which limits the precision of event frequency estimates compared with randomized controlled trials. Finally, the follow-up period of 12 months remains too short to assess long-term kidney outcomes such as sustained eGFR slope or kidney failure, and future analyses with extended observation will be required.

Despite these limitations, the study has important strengths: our study reflects contemporary, guideline-aligned real-world nephrology practice, integrating sparsentan into the therapeutic landscape defined by the KDIGO 2025 framework. Histologic data with Oxford MEST-C scoring were available for the vast majority of patients, enabling a nuanced assessment of structural severity. The cohort’s composition—inclusion of patients across tertiary and private nephrology settings, with histologic characterization and complete background therapy—enhances the generalizability of our findings.

### Conclusions and outlook

In summary, our 12-month real-world data demonstrate that sparsentan, on top of SGLT2i, is associated with a robust and sustained reduction in proteinuria, stable eGFR, and good tolerability in high-risk IgAN patients. These findings reinforce the KDIGO 2025 positioning of sparsentan as a cornerstone of the CKD management pillar. At the same time, emerging anti-inflammatory and immunomodulatory evidence from SPARTAN and the growing experience with dual-pillar combinations such as sparsentan plus TrF-budesonide highlight a paradigm shift towards a combination therapy.

## Supplementary Material

sfag181_Supplemental_Files

## Data Availability

The data underlying this article will be shared on reasonable request to the corresponding author after internal board review.
