# Supplementary material for: Long-term real-world evidence of sparsentan efficacy in patients with IgA nephropathy treated with SGLT2 inhibitors
Source: Clin Kidney J. 2026 Jun 1;19(7):sfag181. doi: 10.1093/ckj/sfag181 (PMC13344172; doi:10.1093/ckj/sfag181)

**Supplemental Appendix**

**Supplemental Tables**

**Table S1.** Comparison of key baseline variables and adverse events between completers and non-completers of the 12 month follow-up.

|  | Completers | Non-completers | p-value |
| --- | --- | --- | --- |
| Baseline variables Median (IQR) | N=17 | N=6 |  |
| Age (y) | 38 (27-47) | 36 (24-62) | 0.99 |
| Gender w (n, %) | 8 (47) | 2 (33) | 0.66 |
| Steroid history (n, %) | 10 (59) | 3 (50) | >0.99 |
| eGFR (CKD-EPI) (mL/min/1.73m²) | 48 (34-66) | 33 (27-76) | 0.35 |
| UPCR (g/g) | 1.55 (0.90-1.85) | 1.66 (1.30-2.34) | 0.52 |
| IFTA | 0.17 (0.10-0.33) | 0.16 (0.09-0.26) | 0.82 |
| fsGS | 0.17 (0.11-0.25) | 0.00 (0.00-0.17) | 0.05 |
| gGS | 0.25 (0.13-0.47) | 0.24 (0.08-0.46) | 0.81 |
|  |  |  |  |
| Adverse events (n, %) | 8 (47) | 3 (50) | >0.99 |
| Hypotension | 2 | 0 |  |
| Mild hyperkalemia (≤5.5 mmol/L) | 1 | 1 |  |
| Oedema | 1 | 1 |  |
| Dizziness | 1 | 0 |  |
| Elevation of liver enzymes | 0 | 1 |  |
| Headache | 1 | 0 |  |
| Pruritus | 1 | 0 |  |
| Gout | 1 | 0 |  |
| Serious adverse events (n, %) | 1 (6) | 0 (0) | >0.99 |
| Pneumonia | 1 | 0 |  |

Abbreviations: BL: Baseline; CKD-EPI: Chronic Kidney Disease Epidemiology Collaboration; eGFR: estimated glomerular filtration rate; fsGS: Focal-segmental glomerulosclerosis; gGS: Global glomerulosclerosis; IQR: Interquartile range; UPCR: Urine protein-creatinine ratio.

**Table S2.** Historic slope analysis cohort and concomitant therapy with TrF-budesonide and SGLT2 inhibitors.

|  | n |
| --- | --- |
| Entire follow-up cohort | 17 |
| Excluding patients with TrF-budesonide^*^ | 7 |
| Remaining patients for historic slope analysis | 10 |
| Patients with eGFR values before SGLT2i initiation | 1 |
| One historical eGFR value obtained before SGLT2i initiation was excluded;  no patient was excluded for this reason. | |
| Patients in historic slope analysis | 10 |
|  |  |

Abbreviations: eGFR: estimated glomerular filtration rate; TrF: Targeted-release formulation.

^*^within 12 months before sparsentan start including those with overlapping therapy.

|  | >12 months | <12 months | ongoing |
| --- | --- | --- | --- |
| History of corticosteroid therapy, n (%) |  |  |  |
| Systemic | 4^+^ (24) | 0 (0) | 0 (0) |
| TrF-budesonide | 0 (0) | 3 (28) | 4 (24) |
|  |  |  |  |
| Time between corticosteroid termination and sparsentan initiation (m) Median (IQR) |  |  |  |
| Systemic | 24 (23-57) | - | - |
| TrF-budesonide | - | 3 (3-4) | -1 (-2 to -1) |
|  |  |  |  |
| Therapy duration (m), Median (IQR) |  |  |  |
| Systemic | 14 (10-22) | - | - |
| TrF-budesonide | - | 7 (7-8) | 9 (8-9) |

**Table S3.** Detailed breakdown of past (longer than 12 months or in the past 12 months before sparsentan initiation) and ongoing corticosteroid treatment: Specification of quantity, time between corticosteroid discontinuation and sparsentan initiation and duration of therapy depending on the type of treatment (systemic or TrF-budesonide).

Abbreviations: IQR: Interquartile range; TrF: Targeted-release formulation; m: month; wk: Week.

^+^one patient received ongoing TrF-budesonide with history of systemic corticosteroid history.

**Table S4.** Adverse events possibly related and unlikely related to sparsentan treatment.

| Event | n (%) |
| --- | --- |
| Possibly related to sparsentan treatment |  |
| Hypotension | 2 (12) |
| Mild hyperkalaemia (≤5.5 mmol/L) | 2 (12) |
| Oedema | 2 (12) |
| Dizziness | 1 (6) |
| Elevation of liver enzymes | 1 (6) |
| Headache | 1 (6) |
| Pruritus | 1 (6) |
| Unlikely related to sparsentan treatment |  |
| Gout | 1 (6) |
| Hospitalisation for pneumonia (SAE) | 1 (6) |
| Abbreviations: SAE: Serious adverse event. |  |

**Supplemental Figures**

**Figure S1.** Overview of corticosteroid therapy (among patients receiving corticosteroids, all received TrF-budesonide) in the year before (<12 months) and during (ongoing) the start of sparsentan.

Abbreviations: TrF: Targeted-release formulation.

**Figure S2.** Course of proteinuria (UPCR) under therapy with sparsentan: (**A**) excluding patients with ongoing corticosteroid therapy, and (**B**) with ongoing corticosteroid therapy AND in the year before (<12 months) compared to the entire cohort (**C**). Box: Median, IQR; Whisker: Min-Max.

Abbreviations: BL: Baseline; eGFR: estimated glomerular filtration rate; IQR: Interquartile range; M: month; TrF: Targeted-release formulation; UPCR: Urine protein-creatinine ratio.

**Figure S3.** Course of haematuria under sparsentan therapy for the entire cohort (**A**) and subgroup without (**B**) and with (**C**) prior/overlapping corticosteroid therapy (TrF-budesonide). 76.5% had a follow-up measurement at the 12-month time point; the remaining patients had their last available measurement at a median of 7.5 month (range 6-8 months).

**Figure S4.**

**A.** Individual eGFR slope trajectories before and after treatment initiation. Each line represents an individual patient. Annualized eGFR slopes were calculated for the 12-month period prior to treatment initiation (historical slope) and for the on-treatment period from week 6 onward (chronic slope). Slopes are connected at the time of treatment initiation (month 0).

Patients are displayed on vertically offset tracks for visualization purposes; vertical displacement reflects the magnitude and direction of eGFR slope (mL/min/1.73 m²/year), but does not represent absolute eGFR values. Patients who received corticosteroid therapy (targeted-release budesonide) during the historical slope period were excluded (n=7), as treatment-associated increases in eGFR may confound slope estimation and limit its validity as a measure of underlying disease progression.

**B.** Individual eGFR slope trajectories before and after treatment initiation for the entire cohort (n=17) including those with corticosteroid therapy during the historical slope period.

Abbreviations: eGFR: estimated glomerular filtration rate.

**Figure S5.** Exploratory subgroup analyses of proteinuria response and remission under sparsentan therapy.

**A.** Median UPCR reduction (%) from baseline to 12 months across various clinical subgroups (age, sex, BMI, baseline UPCR, blood pressure, prior corticosteroid therapy, and haematuria status). Subgroups were divided at median or otherwise clinically indicated.

**B.** Comparison of median UPCR reduction (%) across histologic subgroups (Oxford MEST-C and chronicity indices).

**C.** Proportion of patients achieving complete or partial remission of proteinuria at 12 months, stratified by baseline characteristics. Complete remission (CR) was defined as a UPCR <0.3 g/g; partial remission (PR) was defined as <1.0 g/g, or <0.75 g/g if baseline was between 0.75-1.0 g/g.

Abbreviations: BMI, body mass index; CR, complete remission; PR, partial remission; UPCR, urine protein-creatinine ratio; IFTA, interstitial fibrosis/tubular atrophy; MEST-C, mesangial hypercellularity (M), endocapillary hypercellularity (E), segmental sclerosis (S), tubular atrophy/interstitial fibrosis (T), crescents (C).

**Figure S6.** Exploratory subgroup analyses of proteinuria (UPCR) under therapy with sparsentan. **A**. Patients with vs. without a history of arterial hypertension;

**B.** patients with vs. without history of corticosteroid therapy (including systemic corticosteroids and targeted-release budesonide) at any time point;

**C.** patients with baseline eGFR ≥45 vs. <45 mL/min/1.73 m²; and

**D.** patients with Oxford T score T0 vs. ≥T1. Box: Median, IQR; Whisker: Min-Max.

Abbreviations: BL: Baseline; CS: Corticosteroids; eGFR: estimated glomerular filtration rate; IQR: Interquartile range; UPCR: Urine protein-creatinine ratio; M: month.

**Figure S1**

**
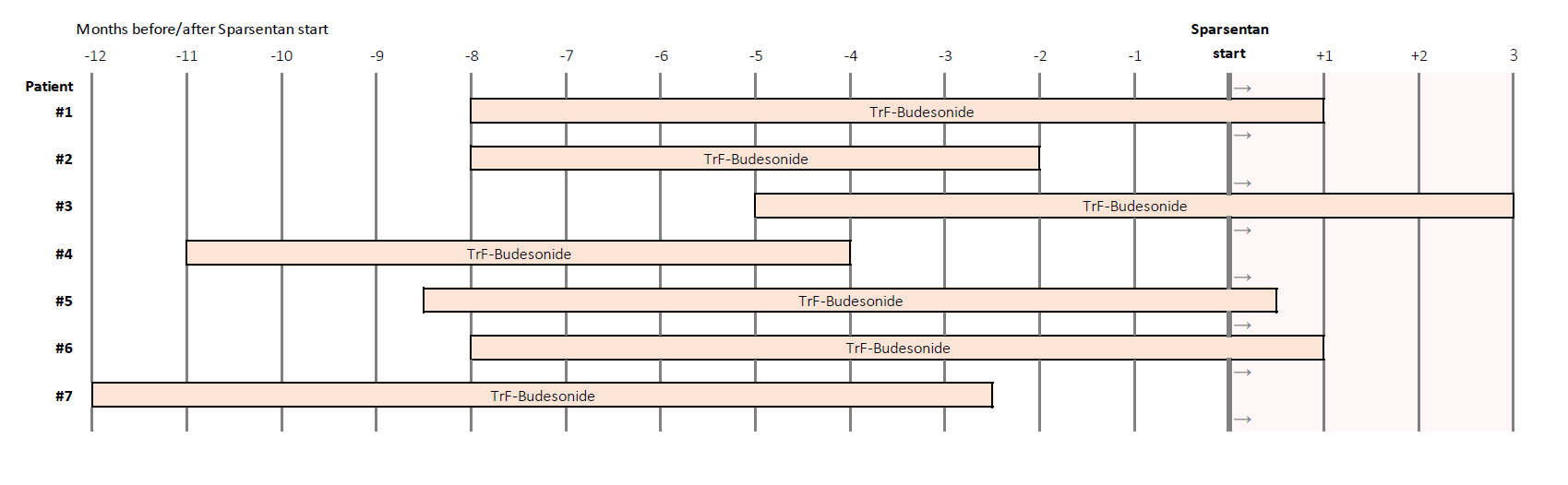
**

**Figure S2**


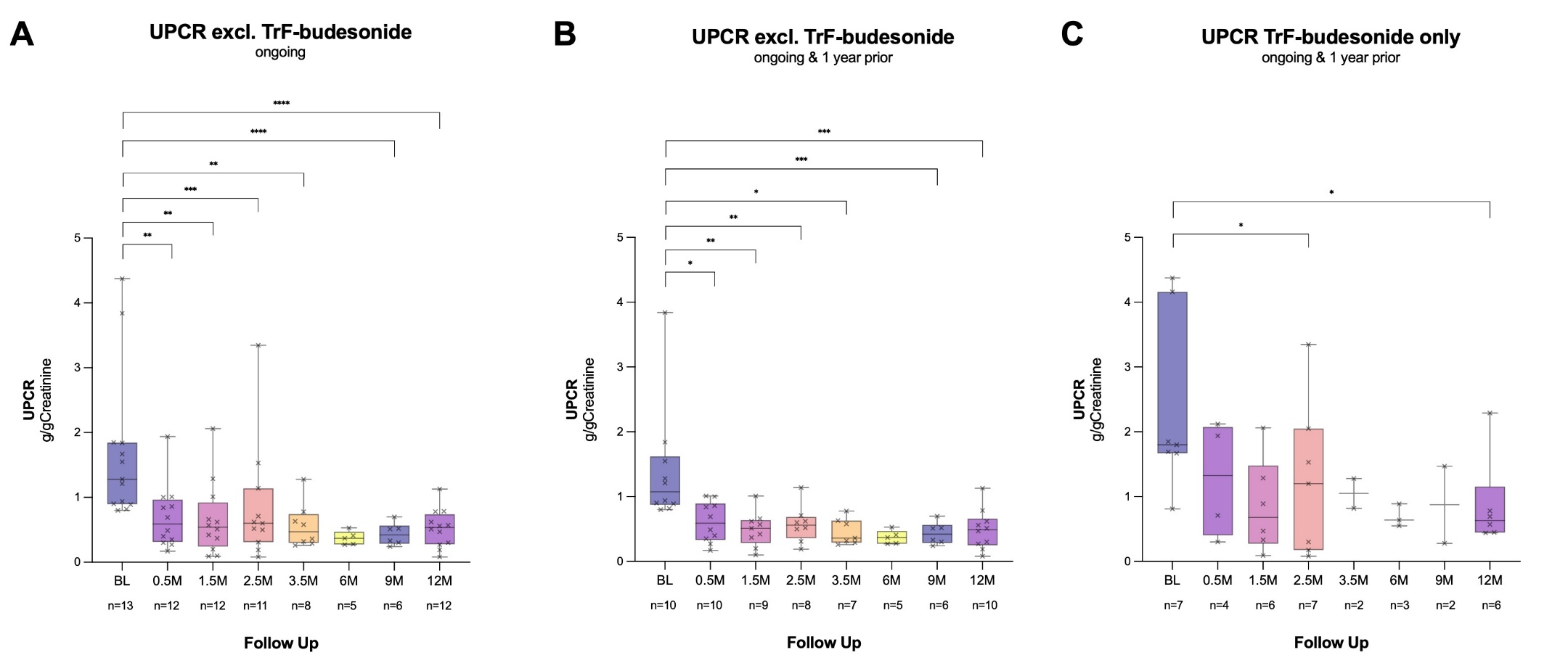


**Figure S3**


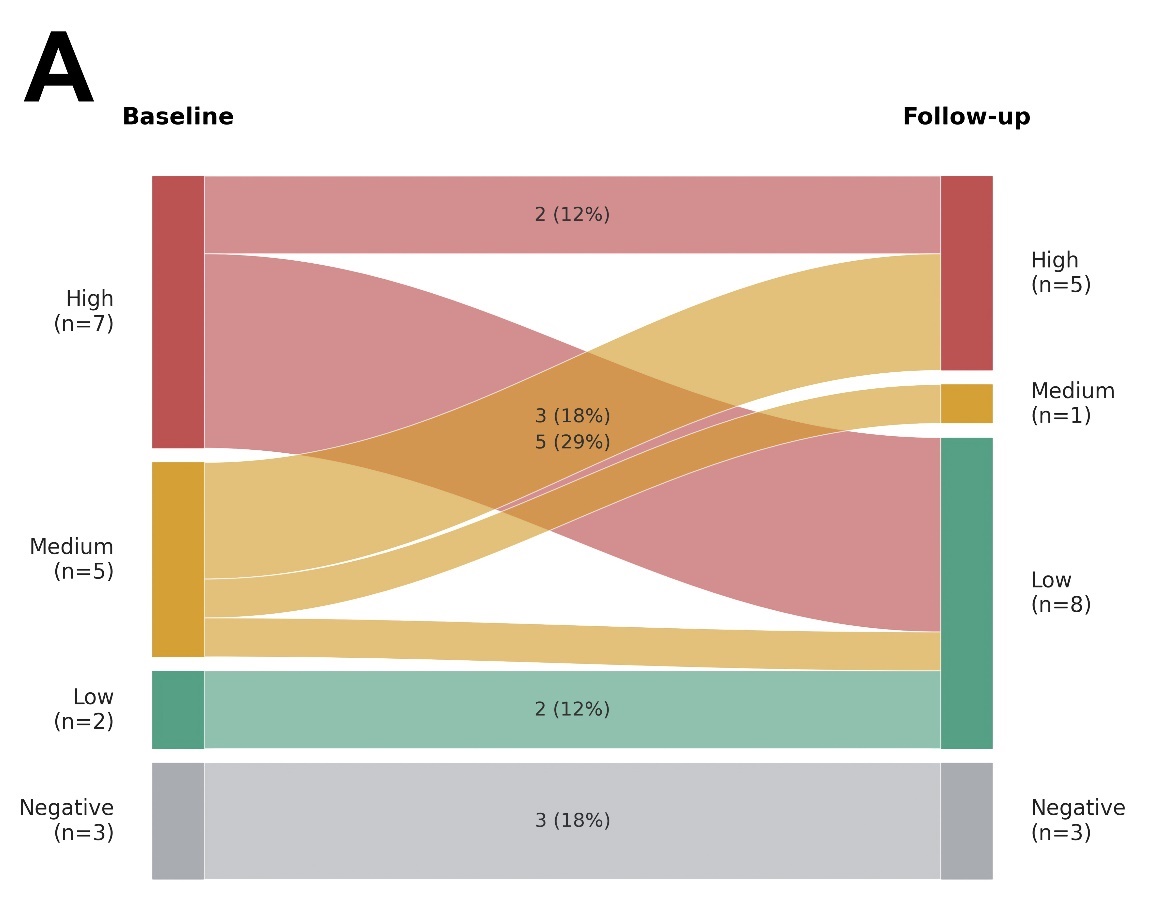


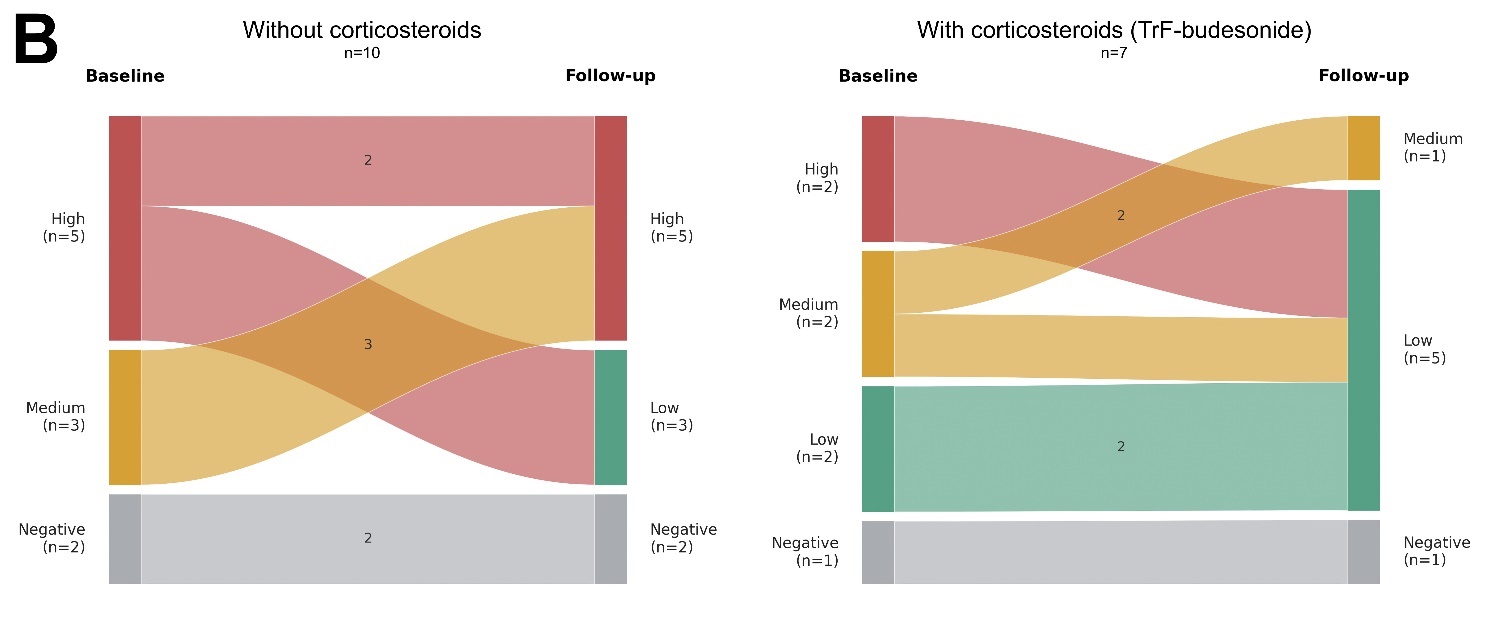


**Figure S4**


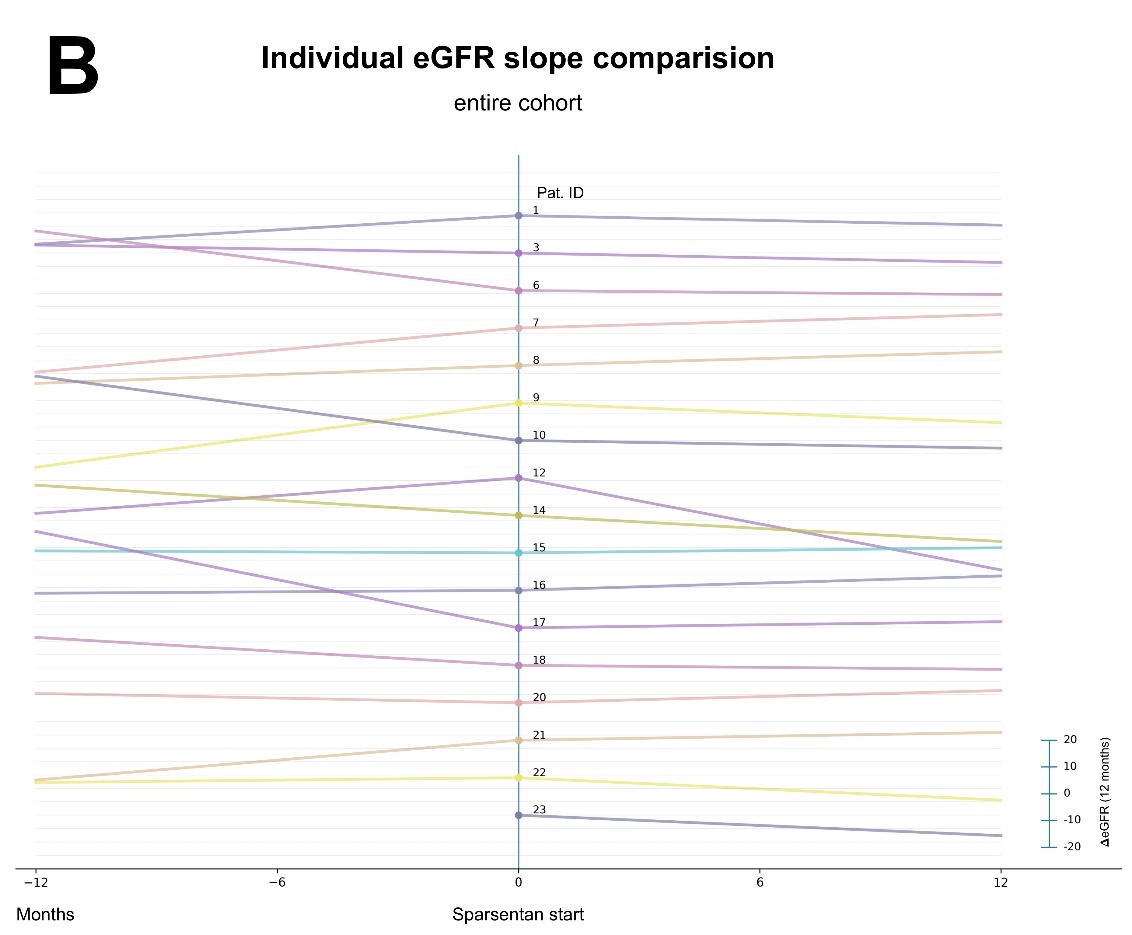

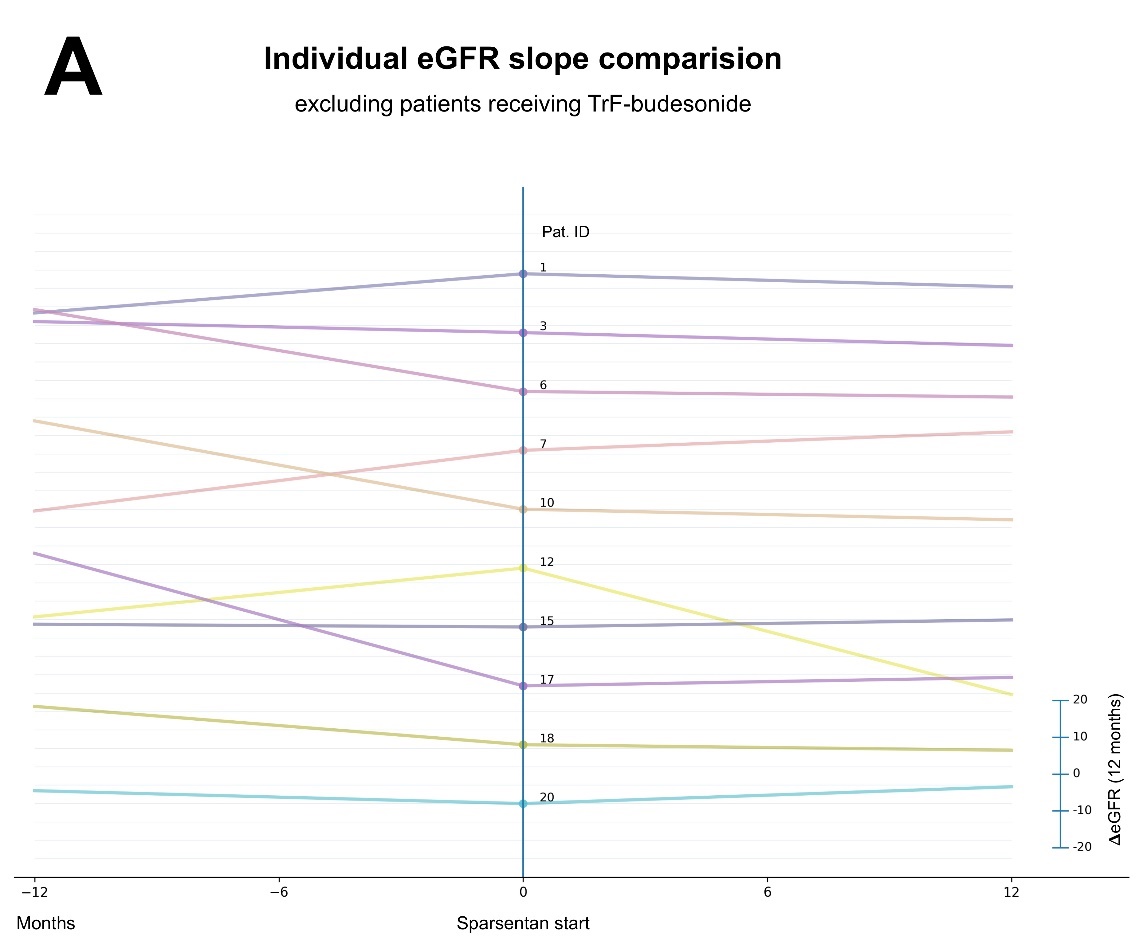


**Figure S5**

**
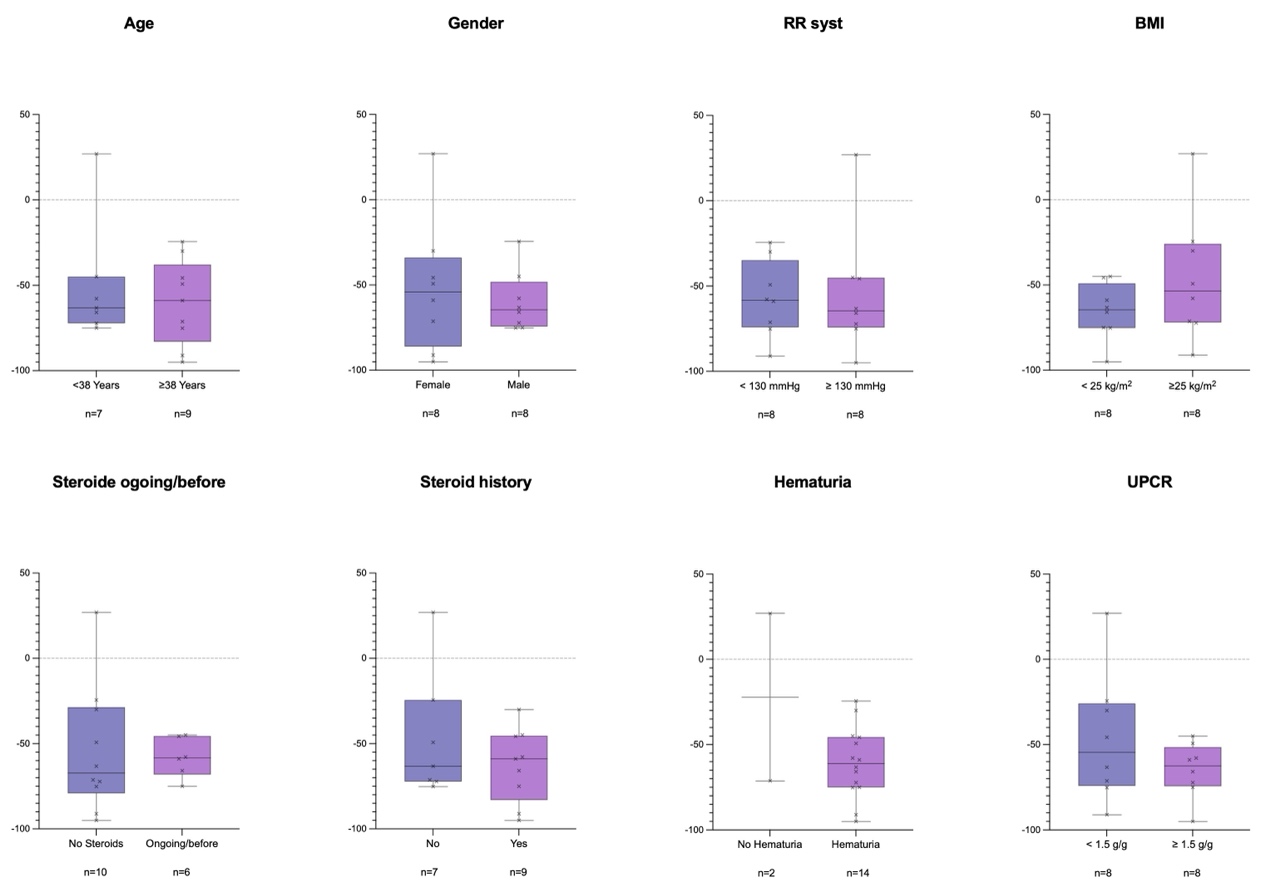
A**

**
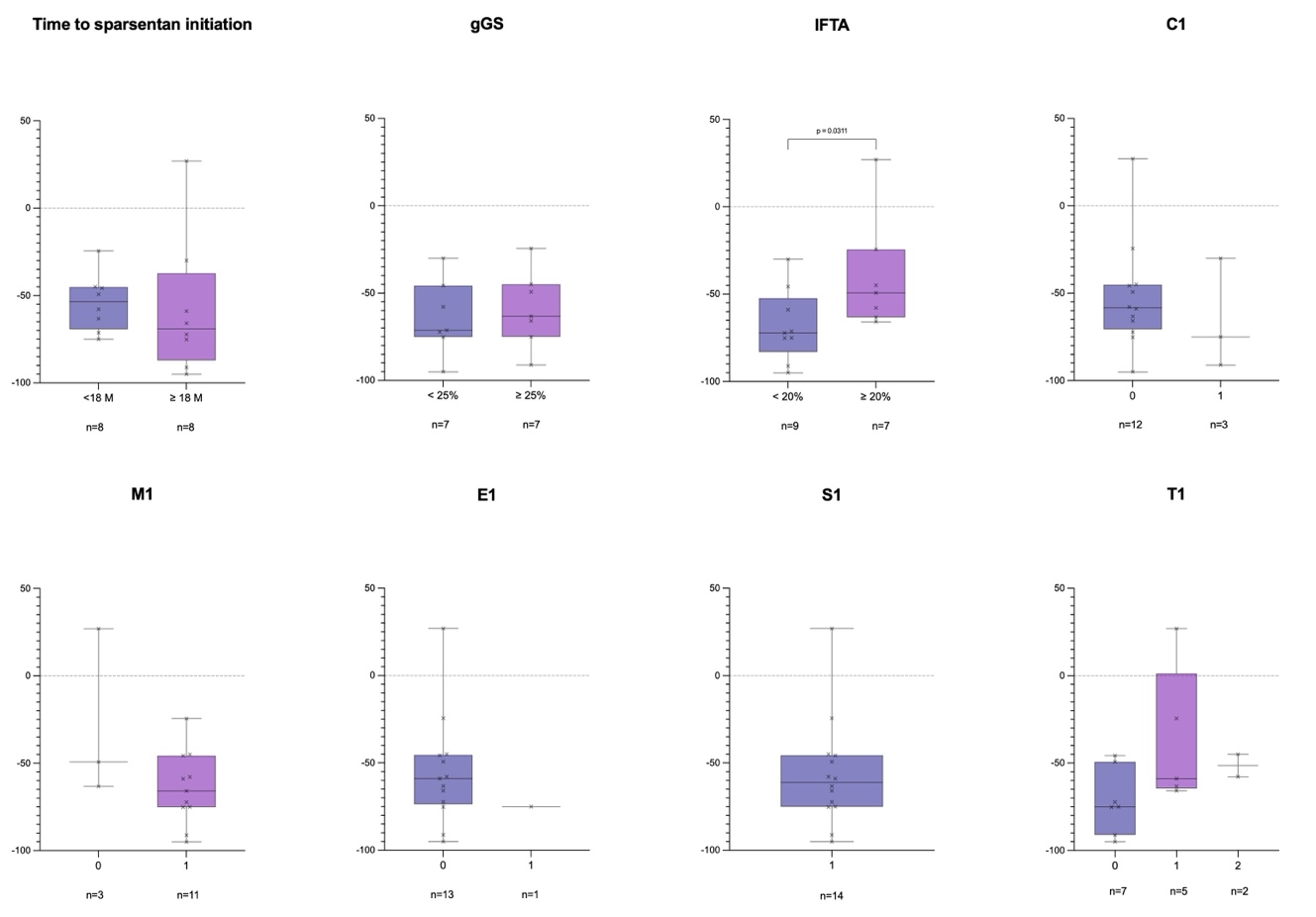
B**

**
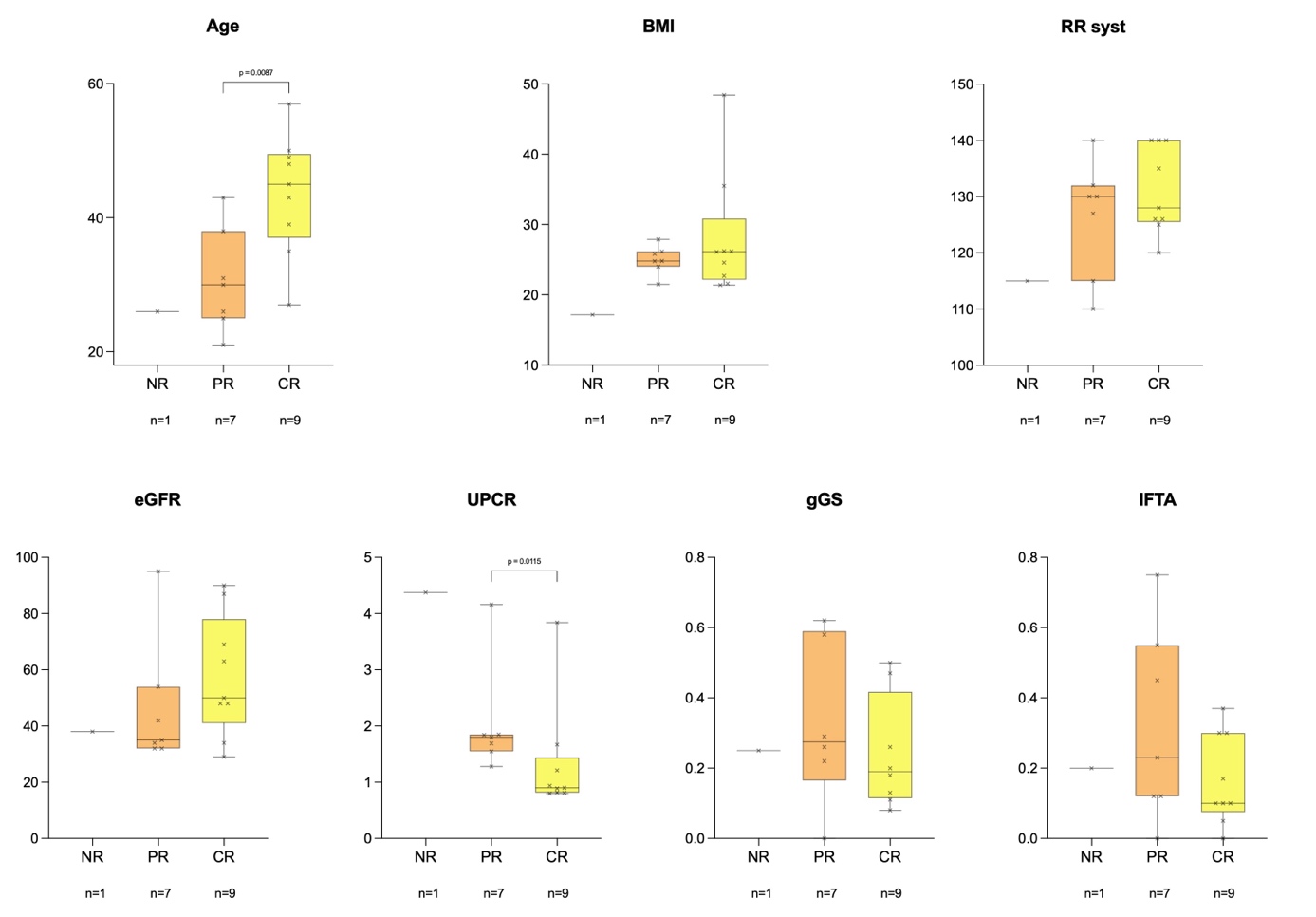
C**

**Figure S6**


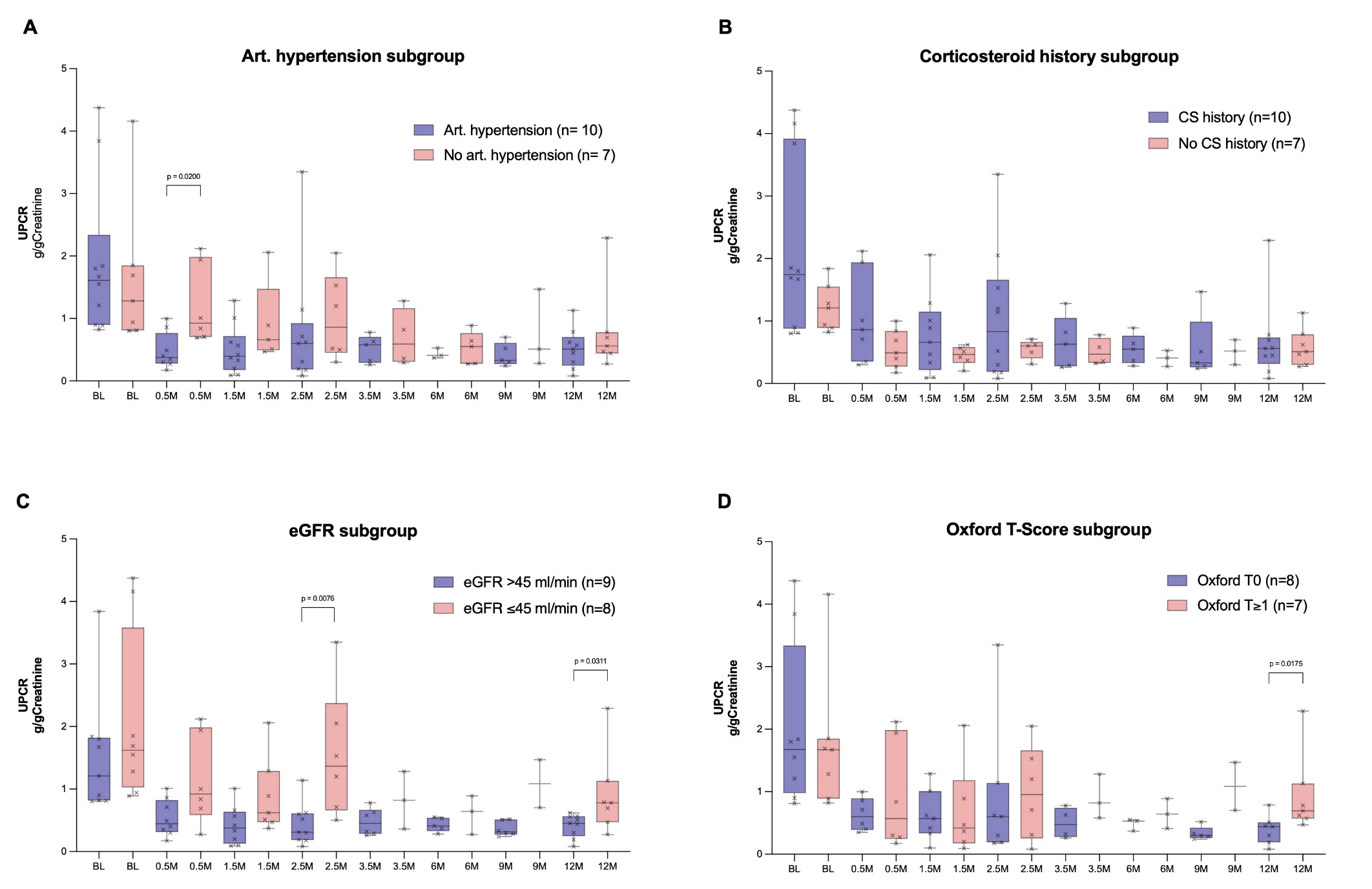

Supplement: sfag181_Supplemental_Files [file sfag181_supplemental_files.zip › 317 Supplemental Appendix.docx]
