# Supplementary figures and images for: Long-term real-world evidence of sparsentan efficacy in patients with IgA nephropathy treated with SGLT2 inhibitors
Source: Clin Kidney J. 2026 Jun 1;19(7):sfag181. doi: 10.1093/ckj/sfag181 (PMC13344172; doi:10.1093/ckj/sfag181)

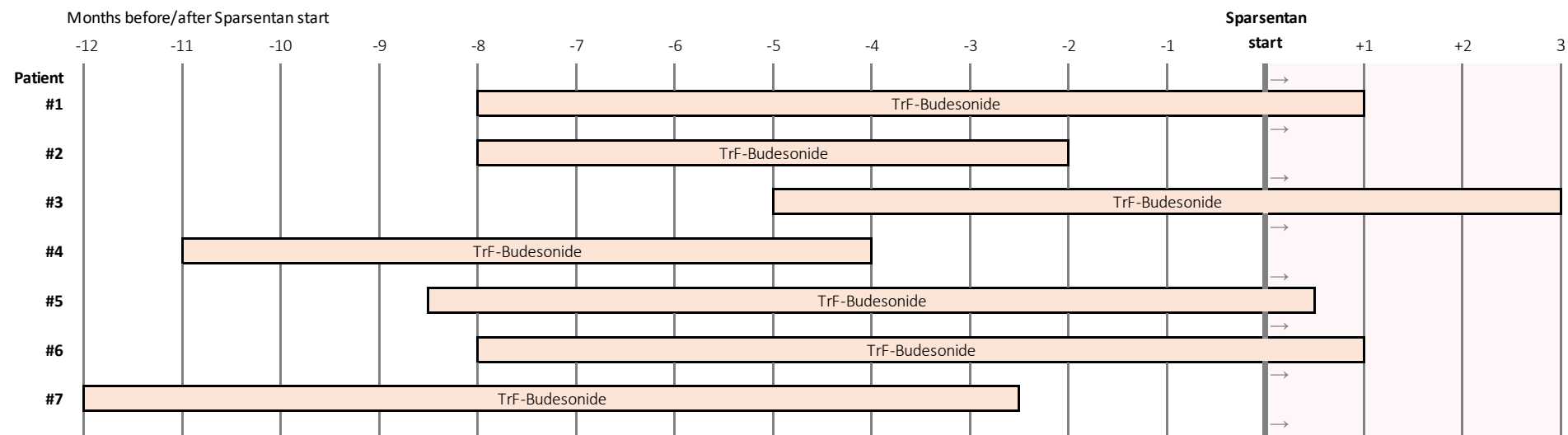

Supplement: sfag181_Supplemental_Files [file sfag181_supplemental_files.zip › FU_Figure_S1.pdf]

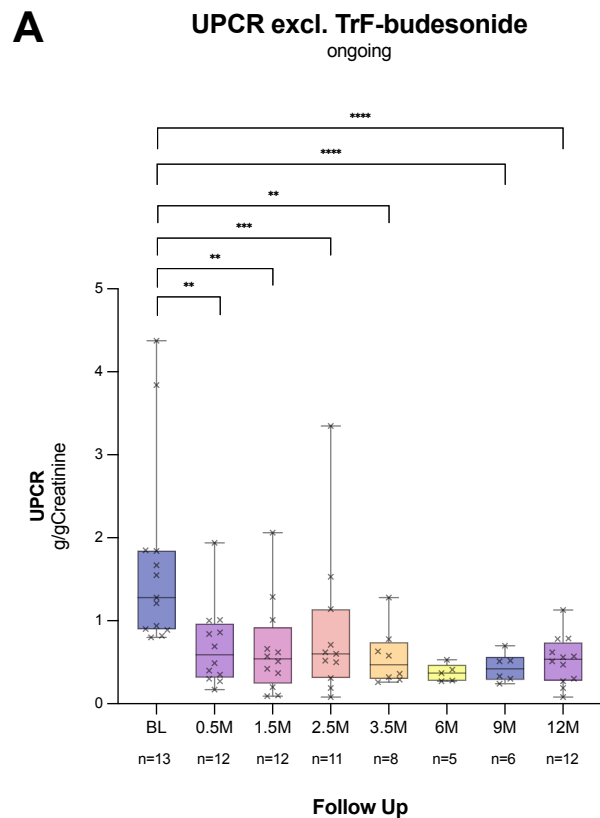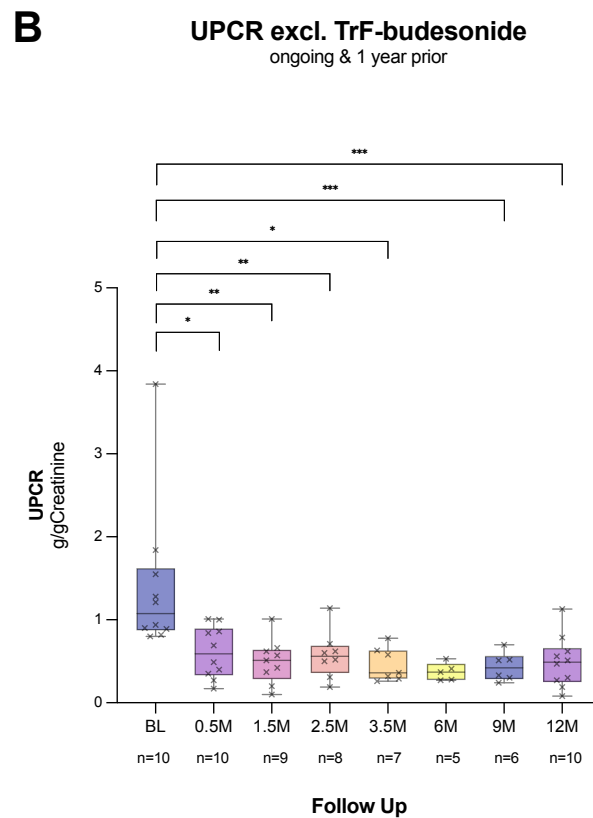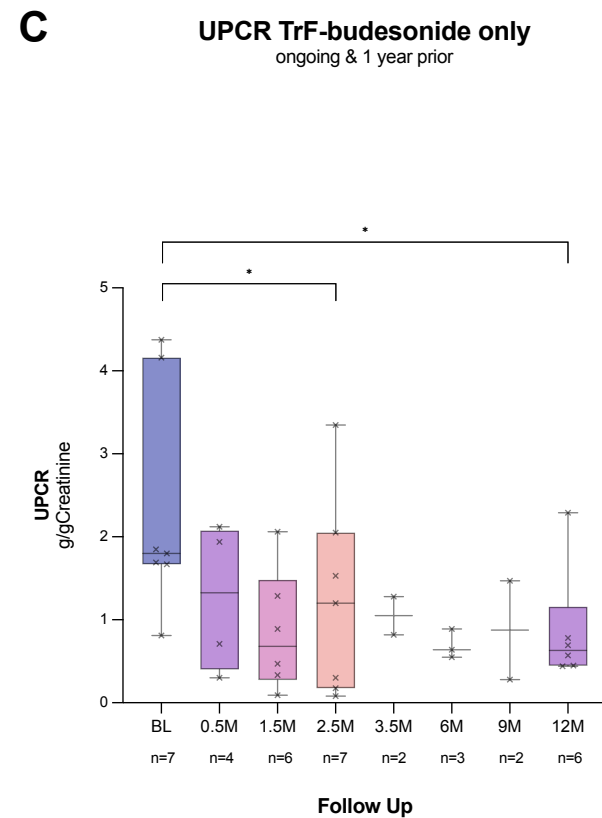

Supplement: sfag181_Supplemental_Files [file sfag181_supplemental_files.zip › FU_Figure_S2.pdf]

# A

## Individual eGFR slope comparision

excluding patients receiving TrF-budesonide

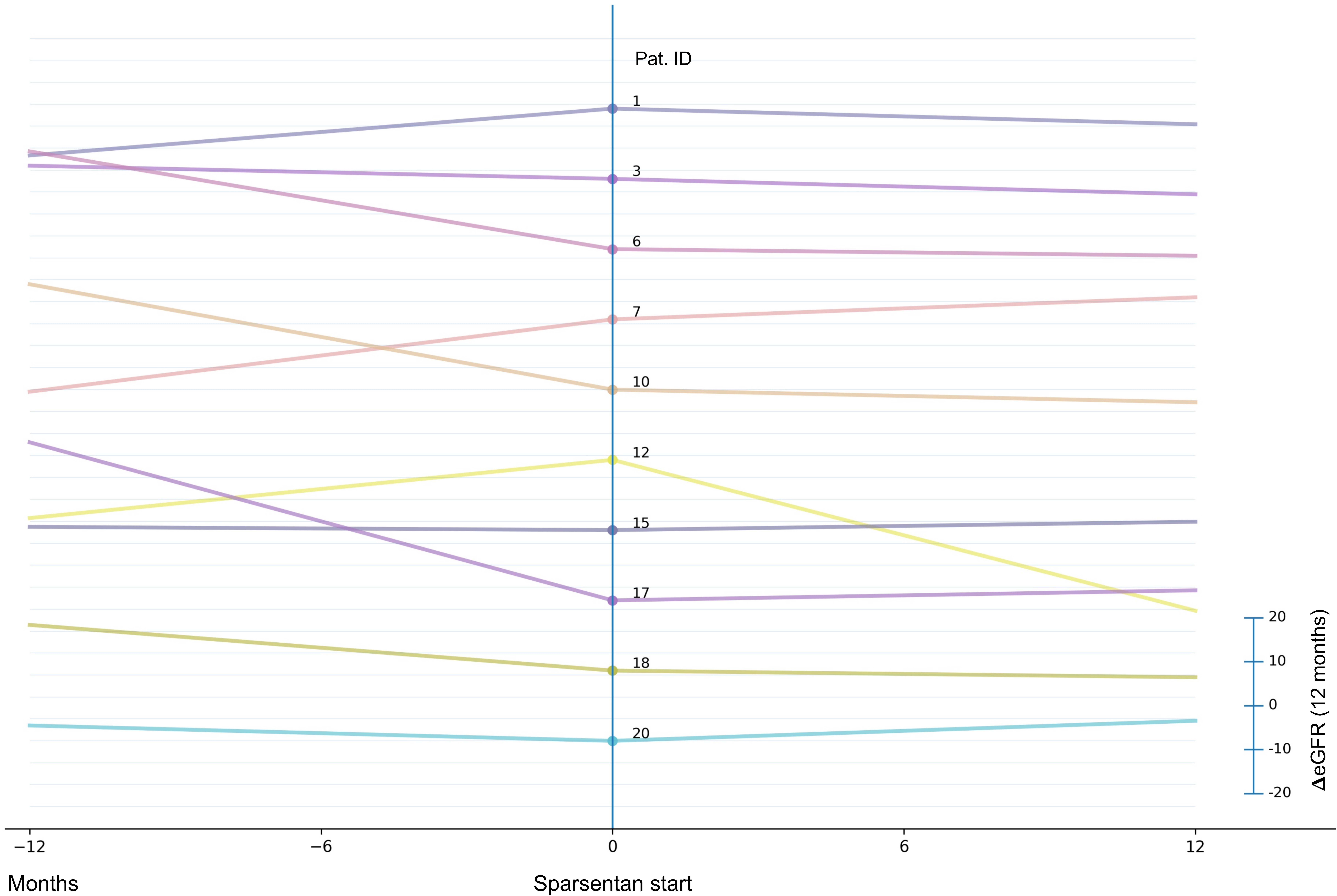

Supplement: sfag181_Supplemental_Files [file sfag181_supplemental_files.zip › FU_Figure_S4A.pdf]

B

# Individual eGFR slope comparision

entire cohort

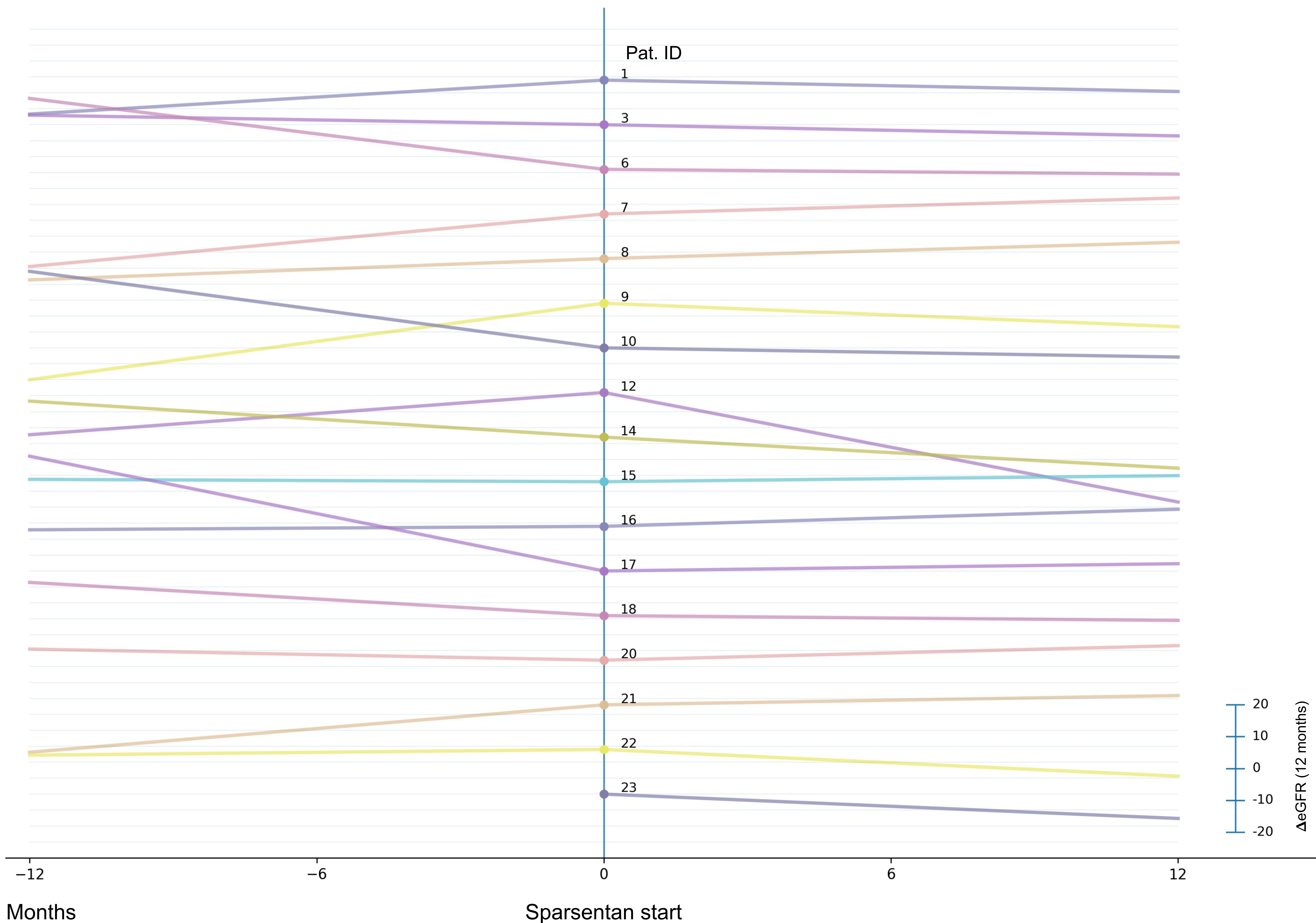

Supplement: sfag181_Supplemental_Files [file sfag181_supplemental_files.zip › FU_Figure_S4B.pdf]
