# Supplementary material for: Long-term real-world evidence of sparsentan efficacy in patients with IgA nephropathy treated with SGLT2 inhibitors
Source: Clin Kidney J. 2026 Jun 1;19(7):sfag181. doi: 10.1093/ckj/sfag181 (PMC13344172; doi:10.1093/ckj/sfag181)
Supplement: sfag181_Supplemental_Files [file FU_Figure_S3A.pdf › FU_Figure_S3A.pdf]

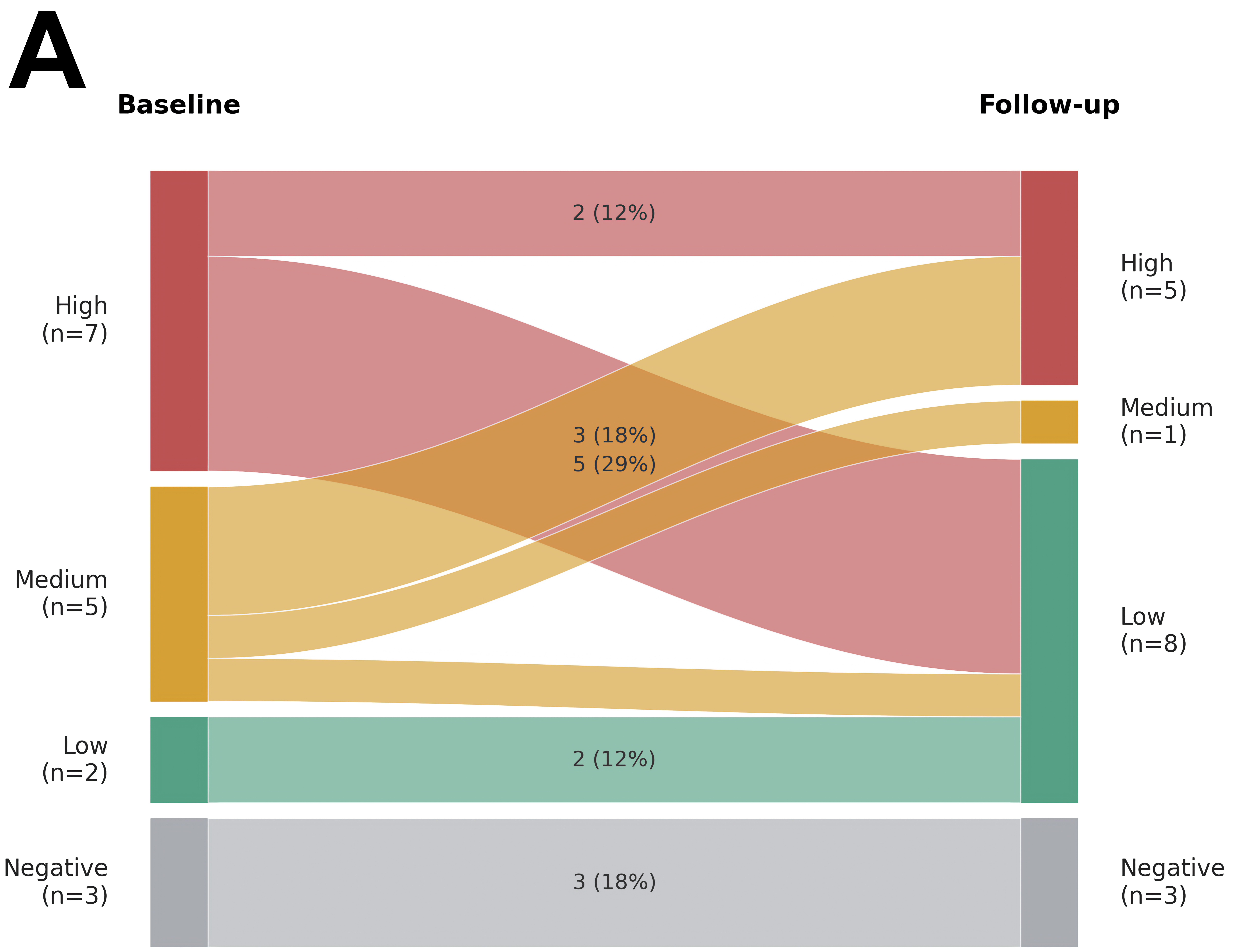
A
Baseline
Follow-up
High
(n=7)
Medium
(n=5)
Low
(n=2)
Negative
(n=3)
2 (12%)
3 (18%)
5 (29%)
2 (12%)
3 (18%)
High
(n=5)
Medium
(n=1)
Low
(n=8)
Negative
(n=3)
